# Energy Drinks and Sports Performance, Cardiovascular Risk, and Genetic Associations; Future Prospects

**DOI:** 10.3390/nu13030715

**Published:** 2021-02-24

**Authors:** Jorge Gutiérrez-Hellín, David Varillas-Delgado

**Affiliations:** 1 Exercise and Sport Sciences, Faculty of Health Sciences, Universidad Francisco de Vitoria, 28223 Madrid, Spain; jorge.gutierrez@ufv.es; 2 Research Unit, Faculty of Medicine, Universidad Francisco de Vitoria, Pozuelo de Alarcon, 28223 Madrid, Spain

**Keywords:** energy drinks, sports performance, caffeine, taurine, health risk, genetics

## Abstract

The consumption of energy drinks (e.g., containing caffeine and taurine) has increased over the last decade among adolescents and athletes to enhance their cognitive level and improve intellectual and athletic performance. Numerous studies have shown that drinking moderate doses of such drinks produces beneficial effects, as they considerably boost the sporting performance of elite athletes in various sports, including both endurance and explosive events. However, apart from their ergogenic effects, the regular consumption of energy drinks also increases blood pressure and consequently incites problems such as hypertension, tachycardia, and nervousness, all of which can lead to cardiovascular disorders. A potential positive correlation between genetics and the moderate consumption of energy drinks and athletic performance has recently been reported; notwithstanding, a better understanding of the genetic variants involved in metabolism is a key area for future research to optimize the dose of energy drink consumed and obtain the maximal ergogenic effect in elite sports. The aim of this literature review, therefore, is to present the results of recent studies, classifying them according to the differences in the associations between energy drinks and: (i) Athletic performance; (ii) cardiovascular risk factors while practicing sports; and (iii) genetic associations and future prospects between the consumption of energy drinks and performance.

## 1. Introduction

Energy drinks first made their appearance in Europe and Asia in 1960. Energy drinks first appeared in Austria in 1987 with a well-known brand and erupted across the globe over the following years. Their consumption has increased exponentially as they have gained in popularity and it has now become a multibillion-dollar industry [1].

It is necessary to differentiate between energy drinks and traditional beverages (coffee, tea, isotonic, hypotonic and hypertonic sports drinks, and soft drinks such as cola). Energy drinks have a high caffeine content which is normally combined with large amounts of vitamins, minerals, taurine, amino acids, and different mixtures of phytochemicals [2].

This type of drink has gained particular prominence, as evidenced by its consumption by various demographic groups, with and without risks of disease, such as youths, workers, students, professional athletes, amateur athletes, and nightlife revelers [3]. No countries restrict or place age limits on the consumption and sale of energy drinks, so they are readily accessible to all populations and ages.

Regarding physical exercise, energy drinks form part of training prioritization in terms of the physical qualities to condition, nutritional practices, pharmacological approach or psychological techniques that can improve training adaptations and/or the output of the exercise [4]. This includes aids that may benefit individuals when exercising, increase the efficiency of the exercise and/or improve subsequent recovery [4,5].

The use of dietary supplements is widespread throughout the general population, but it takes on particular importance for those who practice sports and their consumption by athletes corresponds to a significant proportion of their sales [4,6,7,8]. Dietary supplements can play an important role in helping athletes achieve an ideal intake of calories and nutrients. However, they should never be considered as a substitute for a healthy diet [4].

Energy drinks have emerged as a key dietary supplement to enhance athletic performance, particularly in the acute consumption, with the effects of caffeine and taurine being the most studied in several different sports [9,10,11]. The association between performance and the consumption of energy drinks has been demonstrated in American football and soccer [12,13,14], athletics [15,16,17], volleyball [18], and handball [19], amongst others. Although there is comprehensive evidence of the positive association between the consumption of these drinks and improved sporting performance, there are risks in terms of the potential for cardiovascular problems due to hypertension, altered sleep patterns in adolescents, aggravation of mental illnesses, physiological dependence and an increased possibility of subsequent addiction [20,21], while their potential for toxicity can result in tachycardia, arrhythmia, vomiting, convulsions and even death [22]. The adverse effects of energy drink intake may occur in healthy people, but some people may be particularly prone to complications. High-risk groups include young, caffeine-deficient or caffeine-sensitive pregnant women, competitive athletes, and people with underlying cardiovascular disease [23]. Moreover, the effects of chronic high-dose caffeine and taurine intake in children, adolescents and athletes are not yet known [22].

Possibly one of the most interesting areas for research regarding the consumption of energy drinks associated with athletic performance and the observed cardiovascular risks would be to study the genetic markers that indicate a greater predisposition to improve performance by consuming energy drinks [24] and a protective effect against the damage they can cause to the cardiovascular system. The field of nutrigenomics is expanding our understanding of sports performance [25], but it is still a long way from obtaining evidence-based knowledge. In the area of caffeine, recent evidence related to cytochrome P450 1A2 (CYP1A2) -163C>A polymorphism has helped optimize the caffeine dose an athlete needs to improve their performance [26,27,28] in endurance sports [29], as well as in team sports and explosive efforts [19,28,30,31].

Several studies have shown significant relationships between the consumption of energy drinks, sports performance and increased prevalence of cardiovascular risk factors. However, the results reported to date remain inconsistent, so a full general description of the studies in this field is necessary. For a more detailed analysis of the matter, we have classified recent studies according to the differences in the associations between the energy drinks and: (i) Athletic performance; (ii) cardiovascular risk factors while practicing sports; and (iii) genetic associations and future prospects between the consumption of energy drinks and performance.

## 2. Energy Drinks and Sport Performance

### 2.1. Caffeine and Sport Performance

Energy drinks are beverages that combine different substances among their ingredients, including vitamins, minerals, taurine, amino acids, and mixtures of phytochemicals such as caffeine [2]. Their main impacts on sporting performance are attributed to the effects of caffeine and taurine.

Caffeine (1,3,7-trimethylxanthine) is a phytochemical found in the leaves, fruits, and seeds of various plants such as coffee, tea, and mate [32]. It is a socially-acceptable drug that has been used as an ergogenic aid or performance enhancer in multiple sporting disciplines for many years now. It can be used in various physical forms as a legal nutritional method of improving sports performance in training sessions and competition [33]. Its consumption was banned by the World Anti-Doping Agency (WADA) up until 2004. Athletes were sanctioned if they presented urinary caffeine concentrations above 12 μgm/L. From 2004, caffeine was removed from the list of substances and methods prohibited in- and out-of-competition and placed on the monitoring program [34]. Since then, its sale and consumption has remained unrestricted in the context of international sports.

Caffeine can be consumed in different forms. It is available in gels, bars, chewing gums, lozenges, coffee, tea, cocoa products, cola drinks, and energy drinks. The delivery form determines the rate at which it is absorbed and reaches the bloodstream [35]. The fastest means of caffeine absorption is in the form of chewing gum [36] due to drugs absorbed via the buccal cavity bypass intestinal and hepatic first pass metabolism, which potentially increases their extent of absorption of caffeinated substances. Of all the caffeine consumption routes, the most common method of ingesting caffeine across the globe is in energy drinks [37].

Several researchers have tried to determine the optimal doses necessary to enhance sports performance by studying caffeine dose–response relationships and the subsequent ergogenic effect on athletic performance. In this regard, a randomized study by Pasman et al. [38] analyzed the ergogenic dose–response effect during endurance training in nine well-trained cyclists and observed a significant effect at doses of 5 mg/kg or more (endurance times of 47 ± 13 min, 58 ± 11 min, 59 ± 12 min and 58 ± 12 min for 0 mg, 5 mg, 9 mg and 13 mg caffeine/kg of body weight, respectively [*p* < 0.05]). In a controlled trial, Polito et al. [39] studied the acute effects of ingesting two different doses of caffeine (3 and 6 mg/kg) on performance during a resistance training session (chest press, shoulder press and biceps curl exercises) compared to placebo. The results showed that the placebo group completed significantly fewer repetitions (93.6 ± 22.4) than the groups that took 3 mg/kg (108.0 ± 19.9, *p* = 0.02) and 6 mg/kg (109.3 ± 19.8, *p* = 0.03) of caffeine, while there were no differences between the two caffeine doses. Another controlled study, published by Jenkins et al. [40], compared the dose–response effect of 1, 2 and 3 mg/kg on the athletic performance of 13 trained cyclists against placebo. The results revealed an increase in performance of 4% after ingesting 2 mg/kg and 3% with 3 mg/kg upon completing a 15-min time trail at 60% of VO_2_max.

The literature also includes studies in which the acute consumption of at least 3 mg/kg of caffeine was necessary to improve the performance in different disciplines and movements, as well as cognitively [41]. The optimal dose range to obtain ergogenic effects is 3–6 mg/kg, whether taken acutely in capsules or consumed as an energy drink [16]. Doses of 6 and 9 mg/kg had the same effects without producing an accumulative dose-response effect [42], while the total doses ingested in energy drinks were around 40–325 mg of caffeine, which is comparable to doses of 3–6 mg/kg in capsules [9].

The ergogenic effects of caffeine during exercise were first recorded in the scientific community over 100 years ago [43]. Caffeine is known to have a positive effect by increasing physical performance in endurance sports [44], intermittent exercises such as team and racket sports [45,46], and high-intensity disciplines (from 1–60 min) such as swimming, rowing and middle- and long-distance running [33].

### 2.2. Taurine and Sport Performance

Taurine (2-aminoethanesulphonic acid) is a stimulant commonly found in energy drinks. It is the most prevalent free amino acid found in mammalian muscle tissue [47] and is present in high concentrations in meat and seafood [48].

Taurine corresponds to 50–60% of free amino acids in mammals and fulfils some essential biological functions. Within muscle fiber, taurine stimulates the release of Ca^2+^ from the sarcoplasmic reticulum and maintains the sensitivity of contractile elements to Ca^2+^, acting directly on excitation-contraction-relaxation processes, contractile properties and force production [49], and also exhibiting positive effects on athletic performance in animal models [50]. These findings have been confirmed in both cardiac and skeletal myocytes [51], with such effects considered responsible for the improved performance reported in some studies, such as that of Balshaw et al. [52] who demonstrated the positive effect of the acute consumption of 1000 mg of taurine on sports performance in a 3 km time trial test. A randomized study by Rutherford et al. [53] also observed a 16% increase in fat oxidation during a cycle ergometer test at a moderate intensity (66.5 ± 1.9% VO_2_max) for 90 min in trained cyclists. While another trial attributed prescribed an antioxidant effect to taurine that facilitated the equilibrium in the mitochondrial matrix and improved the efficiency of energy fluctuations in the form of ATP in mice muscle fibers (treatment decreased muscle taurine levels to <40% of controls; *p* < 0.05) [50].

The amounts of taurine in energy drinks range between 71 and 3105 mg [9]. However, some studies have used higher doses (4000–6000 mg/day for 7 days) [54], with the most frequently used doses to date ranging between 1000 and 2000 mg, according to the literature [55].

### 2.3. Energy Drinks Combining Caffeine and Taurine in Sport Performance

Combining caffeine with taurine enhances its effects on the sarcoplasmic reticulum in cardiomyocytes. Both taurine and caffeine have shown in vitro physiological effects on intracellular calcium concentrations in vascular smooth muscle. The highest levels of both substances are found in the heart tissues of patients with congestive heart failure and experimental models of cardiac hypertrophy [56], and can also produce a positive inotropic effect, supporting the idea that caffeine and taurine may act in synergy [56].

The consumption of energy drinks serves different purposes depending on the population drinking them. One of their main uses is to enhance sports performance [57]. This, together with their increased consumption due to their popularity and worldwide availability, has to lead to an increase in the number of scientific studies published in recent years. Various clinical trials have shown that drinking an energy drink that combines caffeine and taurine correlates positively with an improved athletic performance in a range of sporting situations. A study by Quinlivan et al. [58] compared the effect of an energy drink (Red Bull^®^), 3 mg/kg of caffeine and placebo on the sporting performance of cyclists through 1 h time trial at 75% peak power output and found that the group who consumed the energy drink improved their performance by 120 ± 172 s (3768 ± 257 s) (3.1%, *p* = 0.043) compared to placebo (3877 ± 260 s) and the caffeine group (3757 ± 278 s) by 109 ± 153 s (2.8%, *p* = 0.039). Similarly, a double-blind, placebo experimented study presented by Cureton et al. [59] in 16 trained cyclists reported that subjects completed 15–23% more work during a 135 min continuous ride after consuming a caffeinated sports drink with taurine compared to placebo (*p* < 0.05). A controlled trial performed by Ivy et al. [60] in trained cyclists who consumed an energy drink (Red Bull^®^) found that the athletes completed a 1 h time trial at 70% maximum Watts (Wmax) in a shorter time than for placebo (3690 ± 64 s vs. 3874 ± 93 s, *p* < 0.01); which are very similar results to those of Kovacs and Cureton studies [59,61].

In other sporting disciplines with predominantly cyclic and acyclic movements such as football, a randomized study by Lara et al. [13] found that 60 min after drinking an energy drink (Fure^®^: Standardized as 3 mg/kg of body weight, maltodextrin (300 mg/g), taurine (400 mg/g), L-carnitine (40 mg/g), B-group vitamins (10 mg/g) and sodium bicarbonate (100 mg/g)), the athletes’ performance improved compared to placebo in countermovement jumps (heights of 27.4 ± 3.8 cm vs. 26.6 ± 4.0 cm; *p* < 0.05), a 7 × 30 m sprint test (top speeds of 24.5 ± 1.7 km/h vs. 24.2 ± 1.6 km/h; *p* < 0.05) and a simulated football match monitored with GPS devices (total distances run of 7.087 ± 1.501 m vs. 6.631 ± 1.618 m; *p* < 0.05).

In repeated high-intensity efforts, Del Coso et al. [62] also described that the consumption of an energy drink (Fure^®^) increased the athletic performance of elite rugby sevens players as evidenced by greater muscle power output during a 15 s maximal jump test (25.6 ± 11.8 vs. 23.5 ± 10.1 kW with and without the energy drink; *p* < 0.05), running pace during matches (95.4 ± 12.7 vs. 87.5 ± 8.3 m/min, *p* < 0.05) and pace at sprint velocity (6.1 ± 3.4 vs. 4.6 ± 3.3 m/min, *p* < 0.05). In another study, Del Coso et al. [63] also showed the effect of Fure^®^ on athletic performance in sports requiring accelerations and repeated high-intensity movements (elite field hockey players). In this case, the energy drink reduced the distance covered at moderate-intensity running (793 ± 135 and 712 ± 116 m-placebo drink vs. energy drink; *p* = 0.03) and increased the distance covered at high-intensity running (303 ± 67 vs. 358 ± 117 m; *p* = 0.05) and sprinting (85 ± 41 vs. 117 ± 55 m; *p* = 0.02).

A positive correlation between energy drink consumption and improved sporting performance has also been observed in sports such as volleyball. Pérez-López et al. [64] reported a positive correlation between athletic performance and drinking Fure^®^. Compared to placebo, the energy drink increased ball velocity in the standing spike (19.2 ± 2.1 vs. 19.7 ± 1.9 m/s; *p* = 0.023) and jumping spike (17.9 ± 2.2 vs. 18.8 ± 2.2 m/s; *p* = 0.038) and also improved jump height in the squat jump (28.1 ± 3.2 vs. 29.4 ± 3.6 cm; *p* = 0.028), CMJ (countermovement jump) (32.0 ± 4.6 vs. 33.1 ± 4.5 cm; *p* = 0.018), spike jump (43.3 ± 4.7 vs. 44.4 ± 5.0 cm; *p* = 0.025) and block jump (35.2 ± 5.1 vs. 36.1 ± 5.1 cm; *p* = 0.044).

Another randomized study in a sample of sprint swimmers published by Lara et al. [65] revealed a similar effect to that observed in previous works. Consuming Fure^®^ improved the CMJ (49.4 ± 5.3 vs. 50.9 ± 5.2 cm; *p* < 0.05), maximal force during the handgrip test with the right hand (481 ± 49 vs. 498 ± 43 N; *p* < 0.05) and peak power output (273 ± 55 vs. 303 ± 49 W; *p* < 0.05), while reducing the time needed to complete a simulated 50 m competitive swim (27.8 ± 3.4 vs. 27.5 ± 3.2 s; *p* < 0.05).

Furthermore, the effect of energy drink intake has been studied in racket sports such as badminton and tennis. Abian et al. [66] evidenced an ergogenic effect of Fure^®^ consumption on athletic performance in 15 elite badminton players as they increased their squat jump (SJ) height (34.5 ± 4.7 vs. 36.4 ± 4.3 cm; *p* < 0.05), squat jump peak power (*p* < 0.05), CMJ (37.7 ± 4.5 vs. 39.5 ± 5.1 cm; *p* < 0.05) and CMJ peak power (*p* < 0.05) compared to placebo. They also observed more total impacts during a simulated badminton match in comparison with placebo (7395 ± 1594 vs. 7707 ± 2033 impacts; *p* < 0.05). Gallo-Salazar et al. [67] conducted a randomized study that also found a positive correlation between the consumption of energy drinks containing caffeine and taurine and improved athletic performance in elite youth tennis players. Drinking Fure^®^ increased handgrip strength by 4.2 ± 7.2% (*p* = 0.03) in both hands, high intensity running pace (46.7 ± 28.5 vs. 63.3 ± 27.7 m/h; *p* = 0.02), and number of sprints (12.1 ± 1.7 vs. 13.2 ± 1.7; *p* = 0.05) during the simulated match. Compared to the placebo drink, subjects tended to produce a greater maximal running velocity during the sprint test (22.3 ± 2.0 vs. 22.9 ± 2.1 km/h; *p* = 0.07) and win more points on serve (49.7 ± 9.8% vs. 56.4 ± 10.0%; *p* = 0.07) with the caffeinated energy drink. Considering the increased performance in power-specific movements, there is a proven positive correlation between the consumption of energy drinks and improved performance in strength exercises, endurance work, vertical jump tests and specific sprint tests or accelerations (Table 1).

When assessing the effect of consuming energy drinks with caffeine and taurine on the physical component of athletic performance, randomized, double-blind studies show that such consumption correlates positively with aerobic endurance capacity. This was evidenced in treadmill tests conducted by Rahnama et al. [68], who found that VO_2_ max was greater in athletes who ingested energy drinks (*p* < 0.05), as well as in cycle ergometer tests by Ganio et al. [69], in which a group taking caffeine, taurine and B-complex vitamins solution presented improved physical performance between minutes 30 and 120 of the exercise (*p* < 0.001). With respect to the basic physical quality of strength, Sünram-Lea et al. [70] have also shown that the use of 330 mL of energy drinks correlates positively with improvements in isometric strength exercises. Ratamess et al. [71] studied dynamic strength in squat and bench press exercises in subjects given a combination with caffeine, taurine, creatine and essential branched-chain amino acids (BCAAs), reporting statistically significant improvements (*p* < 0.05). In a study by Astley et al. [72], after consuming an energy drink with 64 mg of caffeine and 800 mg of taurine per 200 mL, 15 resistance-trained athletes performed better in specific strength tests compared to placebo, as measured by the number of repetitions in a unilateral knee extension test with the dominant leg (9.5 ± 0.8 vs. 11.5 ± 0.9 reps; *p* = 0.001) and bench presses (8.1 ± 0.5 vs. 10.2 ± 0.4 reps; *p* = 0.01).

Alford et al. [73] have also shown that drinking an energy drink (Red Bull^®^) presents a positive association with athletic performance, reporting significantly (*p* < 0.05) improved aerobic endurance (sustained max. heart rate of 65–75%) and anaerobic performance (sustained max. speed) on cycle ergometers.

In a recent randomized study, Chtourou et al. [74] found that the acute effects of consuming an energy drink included reductions in reaction times, depression, confusion, fatigue, anger, anxiety, rating of perceived exertion (RPE) and affective load scores compared to placebo in 19 physical education students.

However, focusing on jumping movements, results reported in the literature reflect a lesser effect. For instance, the results presented in the Jacobson et al. [75] study, suggest that there is more evidence and a stronger positive association for this type of drink in upper body muscle groups than in large, complex muscle bundles in the lower body.

However, although we have described different studies which demonstrate a positive correlation between the consumption of energy drinks and athletic performance, it is worth highlighting that some studies did not report this positive association (Table 1). A recent study by Thomas et al. [76] investigated the effects of energy drinks on the sporting performance of eSports players (before and after playing three rounds of League of Legends with 15 min recovery between rounds) by assessing attention (Erikson Flanker Test), reaction speed (Go/No-Go test) and working memory (n-back test); only the memory test revealed a statistically significant association (*p* = 0.004), while physical components such as handgrip strength and finger tap speed presented a negative association with sporting performance (*p* = 0.803). Similarly, Umaña-Alvarado and Moncada-Jiménez [77] did not observe a positive effect on physical performance in terms of endurance in 11 runners who competed in two 10 km races, as there were no significant differences between their times when they drank the energy drink or placebo (*p* > 0.05). Candow et al. [78] did not find any differences between an energy drink and placebo among 17 university students after performing a time-to-exhaustion treadmill run at 80% VO_2_max (Red Bull^®^: 12.6 ± 3.8 min, placebo: 11.8 ± 3.4 min; perceived exertion Red Bull^®^: 17.1 ± 2.0, placebo: 16.6 ± 1.8). Nor did Dall’Agnol and Souza [79] report any differences during an incremental exercise test performed in 22 healthy volunteers after they ingested either 160 mg of caffeine with 2000 mg of taurine or placebo (332.50 ± 56.83 vs. 342 ± 40.60 W), while Kammerer et al. [80] did not find any differences in VO_2_max values after volunteer soldiers consumed an energy drink compared to a placebo-controlled scenario. In the same vein, Pettitt et al. [81] observed that consuming an energy drink did not improve the gas exchange threshold in eight recreationally trained subjects during a graded exercise test on a cycle ergometer. Nelson et al. [82] also reported that there was no significant difference in the time-to-exhaustion between placebo and energy drink trials (43.8 ± 9.3 vs. 45.5 ± 9.8 min; *p* = 0.62).

With respect to anaerobic factors, we found three studies that did not evidence a positive association between the consumption of an energy drink and peak anaerobic power (*p* > 0.05) in strength and power athletes, namely those by Hoffman et al. [83], Gwacham and Wagner [12] and Eckerson et al. [84]. Regarding the basic physical quality of muscle strength, a randomized, double-blind study by Goel et al. [85] did not discover any significant differences in maximal voluntary contraction between an energy drink and control (males: Before and after consumption of energy drink 381 ± 37 vs. 371 ± 36 N and control drink 375 ± 61 vs. 363 ± 36 N; females: Energy drink 227 ± 23 vs. 227 ± 32 N and control 234 ± 46 vs. 228 ± 37 N). All detailed results concerning the correlation between energy drinks and sport performance are presented in Table 1.

## 3. Energy Drinks and Cardiovascular Risk Factors

Coffee and caffeine influence the cardiovascular system through their positive inotropic and chronotropic effects, affecting the central nervous system by stimulating locomotor activity and anxiogenic effects. This underlines the need to examine whether these effects may be harmful to health, particularly in the world of sport [86].

Reissig et al. [87] have described several effects linked to excessive caffeine consumption. Over a lifetime, people should only consume large amounts of caffeine for short periods, but this sort of consumption is more common on a regular basis. Furthermore, some people use caffeine to improve their concentration and memory or enhance their physical performance and, in some cases, could develop a dependence syndrome. Caffeine use transforms into “abuse” when individuals develop an uncontrolled need to consume caffeine, even if it is harmful to their health; it transforms into “dependence” when mechanisms of tolerance and abstinence develop, and certain chronic usage habits make caffeine even more damaging. Along with caffeine dependence, subjects who consume extremely high doses continuously for years, ignoring all safety concerns by combining two or more sources of caffeine, for example, coffee and energy drinks, without any evidence that such combinations provide any desirable benefits [86].

Given the significant number of incidents reported among energy drink consumers, it seems pertinent to summarize the available data and establish causal links between the use of these products and the development of health complications. Occasional to moderate consumption of these drinks seems to pose little risk to healthy adults. However, excessive consumption related to their combination with drugs in amounts that far exceed the manufacturers’ recommended intakes could induce negative consequences for human health, especially among subjects with cardiovascular disorders [88].

The risk factors may increase the rate of adverse events, particularly cardiovascular events in individuals who consume energy drinks, due to underlying conditions [37], and they may suffer a caffeine overdose, as has been reported in the literature [22,88,89,90,91]. A lethal dose of caffeine has been noted as 5 g, which is equivalent to approximately 42 cups of coffee with 120 mg caffeine/cup [90]. Sepkowitz et al. [92] suggested that the acute intake of 3 g of caffeine can provoke significant side effects, which may even be fatal, with arrhythmia being the most common factor producing death by this lethal dose. A review by Nawrot et al. [93] stated that a healthy adult can consume up to 400 mg of caffeine/day (equivalent to 6 mg/kg in individuals weighing less than 65 kg) without being associated with any adverse effects.

The combined use of caffeine and ephedra has been reported also as a risk factor for cardiovascular problems [94]. There is evidence to suggest that the short-term use of ephedra, with caffeine, promotes short-term weight loss. One example is the meta-analysis by Shekelle et al. [95] in which subjects who took caffeine and ephedrine lost around 0.9 kg/month more over a short period than the placebo group (*p* < 0.01), no regarding long-term weight loss to support the use of ephedra for athletic performance (*p* > 0.05). Ephedra is known to be ergogenic during anaerobic exercises, such as bench presses (*p* < 0.05), especially when taken with caffeine; however, a point to consider is that systolic blood pressure increased significantly before both tests in subjects treated with ephedrine compared to the other tests [96]. A clinical trial by Haller et al. [97] carried out in 16 healthy subjects showed an increment in the stimulating and metabolic effects of combined ephedrine (25 mg) and caffeine (200 mg) as they increased systolic blood pressure (maximum difference of 11.7 ± 9.4 mmHg compared to placebo; *p* = 0.0005) and heart rate (maximum difference of 5.9 ± 8.8 beats/min; *p* = 0.001). The study demonstrated that, individually, ephedrine and caffeine had modest effects, but in combination, they produced significant cardiovascular, metabolic, and hormonal responses at moderate doses, data which should be taken into account to avoid such risks when indicating the dose to produce the desired ergogenic effect.

## 4. Energy Drinks, Sport Performance, and Genetics

The main enzyme responsible for caffeine metabolism is cytochrome P450 1A2 (CYP1A2), more specifically polymorphism c.-163A>C (rs762551), which corresponds to approximately 95% of caffeine clearance known to date, being known that caffeine metabolism is also carried out by xanthine oxidase and N-acetyltransferase 2 (NAT2) [86,98].

What is more, polymorphism c.1976T>C (rs5751876) in gene ADORA2A (adenosine A2A receptor) has been shown to modulate sleep-wake activity [99], contribute to individual sensitivity to caffeine’s effects on sleep [100], increasing susceptibility to caffeine-induced anxiety [101,102].

In the case of taurine, its effects have been confirmed in animal models. For example, an animal model was used to study the effects of taurine administration on antidepressant-like behaviors in rats and depression-related signal transduction in the hippocampus [103]. Similarly, taurine and β-alanine supplementation were found to be viable therapeutic strategies to improve the fatigue resistance of dystrophic skeletal muscle in mice [104].

Several studies have shown that the rate of caffeine metabolism may have implications for athletic performance, but findings are still currently ambiguous [29,105,106,107]. Some publications and certain sports present ergogenic effects that may help improve performance. For example, Guest et al. [29] conducted 10 km cycling time trials and found that 2 mg/kg of caffeine reduced times by 4.8% (0.8 min) in the AA genotype compared to placebo (17.8 ± 0.4 vs. 17.0 ± 0.3 min; *p* = 0.0005) and 6.8% (1.2 min) at 4 mg/kg (17.8 ± 0.4 vs. 16.6 ± 0.3 min; *p* < 0.0001). However, they did not observe any differences between 2 and 4 mg/kg of caffeine, so the authors suggested that the consumption of 2 and 4 mg/kg improved times in 10 km time trial, but only in AA genotype subjects, while it had no effect on the AC genotype and reduced performance at a dose of 4 mg/kg in the CC genotype. A randomized study by Pataky et al. [107] reported that, besides the genotype shown by CYPA12, the effects of caffeine were associated with the circadian rhythm in a 3 km cycling test. The study revealed that performance was not only influenced by genetic factors at higher doses of 6 mg/kg, along with early activity before 10 a.m., since AC heterozygotes experienced greater performance gains with caffeine ingestion than AA homozygotes, albeit without producing statistically significant results (*p* = 0.12). This shows that factors other than genetic (genotype) and circadian (time of day) parameters affect the ergogenic value of caffeine consumption and may facilitate the more personalized prescription of caffeine ingestion strategies in order to maximize performance. In a randomized study conducted by Womack et al. [105] among trained cyclists administered caffeine or placebo before completing a 40 km time trial, the authors observed a greater reduction in times among caffeine-doped AA homozygotes (4.9%; placebo: 76.1 ± 5.8 min; caffeine: 72.4 ± 4.2 min) compared to C-allele carriers (1.8%; placebo: 72.2 ± 4.2 min; caffeine: 70.9 ± 4.3 min) (*p* < 0.05). These results suggest that homozygous individuals for the A allele of this polymorphism can experience a greater ergogenic effect after ingesting caffeine.

Continuing with more cycling tests in endurance sports, a recent double-blind, crossover study performed by Carswell et al. [108] with dose at 3 mg/kg of caffeine, reported an increase in the cognitive effects for “fast” CYP1A2 metabolizers against “slow” metabolizers with respect to reaction times during exercise (−18 ± 9 vs. −1.0 ± 11 ms), fastest 10% reaction time at rest (−18 ± 11 vs. −3 ± 15 ms) and lapses at rest (−3.8 ± 2.7 vs. +0.4 ± 0.9) (*p* < 0.05), while there were no differences among the ADORA2A genotypes (*p* > 0.05).

Regarding resistance-trained athletes, they can experience acute improvements in resistance exercise, jumping and sprinting performance after consuming caffeine in conjunction with power tests, as shown in the study by Grgic et al. [28] who examined the acute effect of caffeine (3 mg/kg of body mass) compared to placebo. The authors found caffeine enhanced movement velocity and power output across all loads (effect size (ES): 0.20–0.61; *p* < 0.05); the quality and quantity of repetitions completed at 85% of one-repetition maximum (ES: 0.27–0.85; *p* < 0.001); vertical jump height (ES: 0.15; *p* = 0.017); and power output in the Wingate test (ES: 0.33–0.44; *p* < 0.05). However, they did not find a significant interaction effect between CYP1A2 genotype and caffeine intake (*p*-values ranged from 0.094 to 0.994) in the performance results analyzed in this group of athletes.

When analyzing the effects of polymorphism in CYP1A2 and ADORA2A on performance, we start to observe contradictions in team sports, such as the recent study among professional handball players by Muñoz et al. [19], showing that the ergogenic response to acute caffeine intake was not modulated by CYP1A2 or ADORA2A genotypes, with just one genotype x treatment interaction for ball throwing from 7 m (*p* = 0.037), indicating that the ergogenic effect of caffeine in this test was greater in CYP1A2 AA homozygotes than in C-allele carriers. There were no genotype x treatment interactions for either CYP1A2 or ADORA2A in the remaining variables. Collectively, caffeine increased CMJ height, performance in the sprint velocity test and ball throwing velocity from 9 m (2.8–4.3%; *p* = 0.001–0.022; effect size: 0.17–0.31) as a whole group. Similarly, in elite basketball players, Puente et al. [30] found that polymorphism CYP1A2 c.-163C>A had a minimal effect on ergogenic derived from consuming a moderate dose of caffeine (3 mg/kg) compared to placebo. Caffeine only increased Abalakov jump height by a mean of 2.9 ± 3.6% in AA homozygotes (*p* = 0.03), while this result did not reach statistical significance for C-allele carriers (2.3 ± 6.8%; *p* = 0.33), it did not affect sprint time in the CODAT test in either genotype, but it increased the number of impacts during a simulated game in AA homozygotes (4.1 ± 5.3%; *p* = 0.02) and C-allele carriers (3.3 ± 3.2%; *p* = 0.01).

Another randomized study with adolescents (15 ± 2 years) published by Spineli et al. [109] found that a caffeine dose of 6 mg/kg increased number of sit-ups (35 ± 8 vs. 37 ± 9), push-ups (24 ± 11 vs. 26 ± 11) and distance in the Yo-Yo IR1 test (903.2 ± 325.7 vs. 1010.4 ± 378.9 m) (all *p* < 0.05), but did not influence handgrip strength (33.7 ± 8.7 vs. 35.1 ± 8.9 kgf), CMJ (47.9 ± 13.8 vs. 49.3 ± 12.6 cm), spike jump height (52.9 ± 14.5 vs. 54.2 ± 13.6 cm) and time in agility test (15.9 ± 1.3 vs. 15.8 ± 1.1 s) (all *p* > 0.05). The authors concluded that caffeine improves muscular endurance and aerobic performance in adolescent athletes, regardless of their CYP1A2 c.-163 C>A genotype. However, Salinero et al. conducted a randomized pilot study [106] in a healthy population and reported that CYP1A2 variations did not modify the benefits or disadvantages of caffeine during exercise at a dose of 3 mg/kg, as caffeine consumption increased peak power (667 ± 137 vs. 682 ± 140 W; *p* = 0.008) and mean power during the Wingate test (518 ± 111 vs. 527 ± 111 W; *p* < 0.001) without any differences between AA homozygotes and C-allele carriers (*p* > 0.05). The reaction times were similar between caffeine and placebo (269 ± 71 vs. 276 ± 31 ms; *p* = 0.681), again without any differences between AA homozygotes and C-allele carriers (*p* > 0.05). A recent randomized study by Glaister et al. [110] concluded that cyclists who took 5 mg/kg of caffeine achieved a significant time reduction in a time trial (placebo: 30.8 ± 2.3 min; caffeine: 29.7 ± 1.8 min; *p* < 0.05), but there was no effect associated with the genotype for either CYP1A2 or ADORA2A. During submaximal exercise, compared to placebo, caffeine reduced mean heart rate by 2.9 ± 3.7 bpm (*p* < 0.05), with effects that dissipated as exercise intensity increased, although there was no relation to genotype.

One study report on caffeine consumption derived from energy drinks in cyclists, presented by Davenport et al. [111]. They found that a 200 mg supplement of caffeine 35 min before exercise seemed optimal to improve performance in a time trial, reducing the perception of effort in which the individuals with genotype AA of CYP1A2.

Future studies with larger samples are required to fully elucidate this area of research. Table 2 shows the detailed results regarding the correlation between caffeine consumption and its association with genetics.

## 5. Discussion

The results of the studies analyzed herein are ambiguous or even contradictory. This discrepancy is mainly due to the influence of genetics on the ergogenic effects of caffeine, particularly when the correlation between caffeine and athletic performance appears to be more evident and validated in the scientific literature. Due to the lack of studies, there are still some confounding factors which may contribute to these discrepancies in the results.

### 5.1. Energy Drinks and Relationship in Sports Performance

Caffeine is the main component of energy drinks, as mentioned previously. As such, to understand the effects of energy drinks we need to understand the mechanisms that this phytochemical produces in the human body. Caffeine is absorbed rapidly, reaching its peak plasma concentration in 30–120 min [112]. Its absorption rate is regulated by various factors such as the pharmacokinetics in the function of cytochrome activity, mainly those which metabolize caffeine in the liver and above all enzyme CYP1A2. In pharmacological terms, it has been shown that consuming more than 6 mg of caffeine per kg of body mass seems to saturate hepatic caffeine metabolism, as stronger effects are not observed when greater amounts are ingested [113].

The consumption of caffeine stimulates the central nervous system through the blockade of peripheral and cerebral adenosine receptors, thus generating a delay in the onset of fatigue [114]. Caffeine also stimulates motor neurons by increasing their recruitment [115]. Its effect is also associated with an increased release of Ca^2+^ stored in intracellular neuronal reserves, triggering a range of important neuronal processes [116]. Caffeine enhances the mobilization of glycerol and free acids in blood [117], thereby increasing fat oxidation during exercise at low and moderate loads [118]. Furthermore, caffeine stimulates adrenaline (epinephrine) secretion. This response, together with all those mentioned above, produces a series of metabolic changes associated with ergogenic effects that illustrate its consumption in athletic performance [16].

With respect to caffeine’s different mechanisms of action once ingested by humans, its use is considered an ergogenic aid to increase sporting performance [119] and improve cognitive aspects such as concentration, alertness and reaction time [120]. In addition, energy drinks possess effective doses that trigger these stimulating consequences in the human body, so it is not surprising that the consumption of such drinks and their inclusion of taurine and other phytochemicals exhibits a positive correlation with physical performance in many different sporting movements, disciplines and sports (Table 1).

On the other hand, we also found several studies that did not observe a positive association between the consumption of energy drinks combining caffeine and taurine and improved athletic performance, even with the risk they may pose to the nervous system of those who consume them [121]. The reasons why these studies did not evidence any positive associations with increased physical performance could be because their subjects were already habituated to caffeine consumption [78,122], because of the subjects’ high level of training, or due to a lack of statistical power arising from small sample sizes.

A systematic review and meta-analysis [9] were published in 2017 to study the ergogenic effects of energy drinks and different expressions of sporting performance. The authors concluded that the analyzed literature demonstrated a significant improvement in athletic performance in tests of muscle strength, jumps, resistance exercises and specific actions performed in distinct sporting disciplines and that drinks which contained taurine significantly increased these effects. They also highlighted the need for more research into the area and more control in the testing protocols. This review confirms the data published in the literature and completes the findings reported to date.

### 5.2. Energy Drinks and Relationship in Cardiovascular Risk

Adolescents, young adults and above all athletes are drawn towards energy drinks because of their perceived benefits. However, these individuals are often unaware of the harmful effects associated with these drinks. The body gets used to consuming these drinks in order to function and maintain a level of performance [60,84]. Although a plethora of studies have shown that energy drinks are more effective at enhancing cognitive function or increasing energy levels compared to traditional soft drinks, their excessive consumption can be detrimental to both an individual’s athletic performance and their health [85]. The use of energy drinks, coffee and other caffeinated drinks as a substitute for sleep in relation to school, sporting or day-to-day duties has developed into a regular habit among adolescents and young adults, giving rise to particular concern about the risk such consumption could imply for the population’s health [84]. Numerous studies have evidenced the risks of cardiovascular diseases that derive from constant dependence in an otherwise healthy population [86,87], including hypertension, tachycardia and even sudden death from lethal doses of 5 g, equivalent to approximately 42 cups of coffee with 120 mg caffeine/cup [90], or just 3 g [92].

The ergogenic dose of caffeine necessary to improve neuromuscular performance during sport depends on the magnitude and duration of the activity. Researchers have shown that 3 mg/kg is enough to improve muscle actions in strength and endurance sports, yet a higher dose (9 mg/kg) is associated with the appearance of unwanted side effects [123]. At the same time, a healthy adult can consume up to 400 mg of caffeine/day (equivalent to 6 mg/kg in individuals weighing less than 65 kg) without producing any adverse effects [93]. Studies which have reported changes in heart rate and blood pressure involved the supplementation of caffeine with ephedrine in synergy, such as the works of Haller et al. [97] and Shekelle et al. [95], which observed increases in heart rate and blood pressure and weight loss, respectively, but at moderate doses (200 mg of caffeine and 25 mg of ephedrine) and without the participants reporting any adverse events. Similar results were found in the study of Nowak et al. [124], in which, after ingesting energy drinks containing 80 mg of caffeine, glucose, taurine, vitamins and other ingredients glucuronide, acute intake of energy drinks can increase diastolic blood pressure by more than 8%, blood sugar and discomfort level of healthy young people. Besides, Hajsadeghi et al. [125] confirmed that drinking energy drinks (caffeinated energy drinks) before and at a specific time point of 4 h may lead to decreased heart rate and more frequent ST-segment and T-wave (ST-T) changes in healthy young people.

In addition, Red Bull^®^ has the same effect on blood pressure as a considerable amount of caffeine, this increase occurs through different hemodynamic pathways. Reed Bull mainly affects cardiac parameters, while caffeine mainly causes vascular effects. In addition, the auxiliary components of Red Bull^®^ (taurine, glucuronide, and group B vitamins) do not seem to affect these pathways [126].

To date, no studies have recorded any severe adverse events in athletes related to energy drink use, with the doses that are currently used by this population ideal for enhancing sporting performance without provoking harm thanks to the control exerted by the WADA [34].

### 5.3. Energy Drinks, Genetics, and the Relationship with Athletic Performance; Future Prospects

The influence of the genes CYP1A2 and ADORA2A on the body’s response to caffeine has been discussed in detail and there is a general overview in the current literature. The role of these two genes can explain a significant proportion of the interindividual variation in performance following caffeine ingestion reported in studies [26]. By determining the extent to which these genes and any new polymorphisms discovered in the future can moderate an individual’s response to caffeine during exercise, we will be able to guarantee that caffeine supplementation programs can be tailored to each athlete in order to maximize the potential ergogenic effect of energy drinks [27].

Several randomized, placebo-controlled studies have published data that feature subjects’ inhomogeneous groups in terms of sporting level, age and sex for both the caffeine and placebo cohorts [19,28,29,30,105,106,107,108,109,110]. Most studies reviewed in the present work that examined the link between genetics and athletic performance in strength and endurance events observed that polymorphism c.-163C>A (rs762551) in gene CYP1A2 presents an association with improved performance, while they did not observe any ergogenic effects in relation to gene ADORA2A. It has been shown that the most effective dose to improve sports performance in association with genetics is around 3 mg/kg of body weight (equivalent to 200 mg of caffeine per energy drink), as this enhances endurance performance in cycling [29], reduces reaction times and improves cognitive performance [108], and augments power [28], as observed in team sports, such as handball [19] and basketball [30], where improvements in various tests were reported in association with polymorphism of CYP1A2. In turn, a dose of 5–6 mg/kg of body weight (equivalent to 400–500 mg of caffeine), particularly in cycling [105,107,110], is known to prolong the ergogenic effects in certain aspects of endurance events (cycling, athletics, triathlon) and therefore sustain improved performance. Furthermore, genetic factors are known to maintain this performance in competitions, data which should be studied in these events in the future, as explained by Grgic et al. [31]. In their review of the ergogenic effects of caffeine associated with polymorphism in CYP1A2, the authors found few studies that reported a better response to caffeine in terms of sporting performance among the AA genotype subjects, observing that variations in this gene can modular the ergogenic effects of caffeine, but differences between genotypes were small, inconsistent or limited to specific exercises. Grgic’s findings agree with those presented in our review, highlighting areas for future research in order to amplify the information on genetics and improved athletic performance with the use of energy drinks.

Polymorphism c.1976T>C (rs5751876) in gene ADORA2A has been shown to modulate sleep–wake activity [99,100]. Yet when studied in conjunction with a polymorphism in CYP1A2 the results did not reveal a clear relationship with performance, concentration or states of nervousness, as indicated by Carswell et al. [108]. While self-reported insomnia, diuresis and excessive activity was documented in handball players with TT genotype [19] and an ergogenic response to caffeine consumption has recently been observed in C-allele carriers [127].

Successive studies with larger sample sizes should be conducted to address the discrepancies between genetic condition and sports performance that have been exposed in the scientific literature.

According to the recent review of caffeine in the context of athletic performance published by Martins et al. [128] improvements in performance were noted at doses of 2–9 mg/kg. This is notable because the physiological mechanisms that are involved when the dose is increased remain unclear, since the subject’s regular consumption and the time of day when the caffeine is taken may have positive or negative effects on the ensuing benefits. Recent findings show that caffeine can enhance or diminish performance during exercise. These antagonistic responses may even occur when using the same dose and in individuals with the same characteristics. These effects are subdivided into those linked to the caffeine itself, daily consumption habits, physiological factors and genetic factors. A range of hypotheses has been put forward that indicate the genetic influences related to polymorphisms in the genes CYP1A2 and ADORA2A. Current evidence suggests that CYP1A2 only has a strong influence in endurance activities, where the TT genotype has greater benefits upon increasing the caffeine dose, while individuals of CC genotype suffer a decline in performance with the use of caffeine. On the other hand, there do not appear to be any differences between responses with respect to the polymorphism in CPY1A2 in intermittent and anaerobic exercises, but only a few studies with limited participants have been completed, which underlines this is an interesting area for future studies. Another potential focal point where there is a lack of results is the caffeine ingestion time before carrying out short tests [129], with the ergogenic effect of caffeine known to develop 1–3 h after consumption, as highlighted by the present review and in accordance with the 2002 study by Bell et al. [130]. It is important to note that there are only ambiguous results in the current literature concerning physical characteristics (training, sex, etc.), caffeine abstinence and the possible impact of polymorphism in gene ADORA2A. All the factors in this review should be taken into account when designing studies that aim to improve the current body of research, as described in this section on future prospects for an improved understanding of the effects of caffeine, its ergogenic effects and the role of genetics.

Year after year, new polymorphisms are discovered to have a bearing on different aspects of athletic performance, with as many as 120 polymorphisms known to hold a direct relationship with the performance [131]. According to a recent review by Joyner [132], we are still a long way from developing a complete understanding of genetics’ influence on sporting performance, and in the future, we might be able to model it using field tests, which, with more variables, could be highly predictive of an athlete’s performance. In this context, a recent study of interest is that of Varillas et al. [133], which looked at another cytochrome P450 gene (CYP2D6) and variants in the glutathione S-transferase family (GSTM, GSTP, and GSTP), as it found “favorable” frequencies in endurance athletes compared to a control population using genetic scoring, as Joyner explains, to determine which are the most useful in terms of predicting performance. Such information on these hepatic variables would be of interest to promote genetic scoring in various genes involved in hepatic metabolism and which affect caffeine’s action—such as CYP1A2, ADORA2A, CYP2D6, and the glutathione S-transferase (GST) family—to expand on current knowledge and help resolve some of the contrasting results in the literature. Genetic scoring could represent a more powerful tool and means of discovering the weight of each polymorphism on the metabolism of caffeine absorbed from energy drinks and its ergogenic effects in endurance sports and strength tests [31].

## 6. Conclusions

Energy drinks mainly comprised of caffeine and taurine demonstrate a positive effect in terms of enhancing sporting performance, although several studies did not report any such effect. The exact mechanism of caffeine’s ergogenic effect during exercise is still relatively unknown; the same is true for the risks its consumption may pose to the health of athletes, as it can provoke multifactorial adverse events. The field has received insufficient attention and requires further investigation to answer all of these questions. Nevertheless, the increase in athletic performance may also be related to alterations in perceived effort, reaction time, cognition and/or mood. There is a dearth of studies that confirm caffeine’s effectiveness in sports such as sprinting, athletics, football, tennis, handball, and hockey, plus a lack of research on the role of genetics, where the results appear to contradict each other. Therefore, we need to study more cytochromes involved in caffeine metabolism to open new lines in our understanding of the association between genetics and athletic performance following the consumption of caffeine and, in turn, energy drinks.

## Figures and Tables

**Table 1:** Characteristics of studies referring to the association between energy drinks and sport performance.

Statistically Significant Positive Association								
First Author/Country/	Year	Study Design	Sample (n)	Gender/Age/Mean Age	Caffeine Dosage	Taurine Dosage	Exercise Performance Test	Result
Kovacs et at./the Netherlands [61]	1998	Randomized double blind, placebo controlled, crossover	15 (Well-trained triathletes and cyclists)	Male, 23.3 ± 0.9 y	Drinks of 14 mL/kg BM of a placebo and four carbohydrate-electrolyte solution containing 150 mg/L CAF, 225 mg/L CAF, and 320 mg/L CAF	All drinks contained 70 mg/L	Warm-up protocol (20 min) and a 1-h time trial cycling performance	Improved in min with caffeine supplementation: 62.5 ± 1.3, 61.5 ± 1.1, 60.4 ± 1.0, 58.9 ± 1.0, and 58.9 ± 1.2 min for placebo-carbohydrate-electrolyte solution, carbohydrate-electrolyte solution-150mg/L, carbohydrate-electrolyte solution-225mg/L, and carbohydrate-electrolyte solution-320mg/L, respectively (*p* < 0.05)
Alford et al./the UK [73]	2001	Double-blind, randomized, repeated measures	39 (Healthy volunteers)	Both, 18–30 y	Red Bull^®^ Energy Drink contains carbonated water, caffeine (80 mg)	Red Bull Energy Drink contains taurine (1000 mg)	Psychomotor performance (reaction time, concentration, memory), subjective alertness and physical endurance on cycle ergometer	Red Bull^®^ Energy Drink significantly improved (*p* < 0.05) aerobic endurance (maintaining 65–75% max. heart rate) and anaerobic performance (maintaining max. speed)
Cureton et al./the USA [59]	2007	A double-blind, placebo-controlled, repeated-measures experimental design	16 (Trained cyclist)	Male, 27 ± 7 y	Commercially available 7% CHO-electrolyte sports drink containing 195 mg/L caffeine	1.92 g/L taurine	Cycled continuously for a total of 135 min, alternating the exercise intensity between 60% and 75% VO_2_max every 15 min for the first 120 min. The last 15 min of cycling, the subjects were instructed to ride as hard as possible	The performance ride was 15–23% greater for energy drink compared to placebo. Ratings of perceived exertion were lower with energy drink. Strength loss was less for energy drink than for the other beverages or placebo (5% vs. 15%)
Ratamess et al./the USA [71]	2007	Randomized, double-blind crossover	8 (Resistance trained)	Male, 20 ± 2 y	Amino Shooter, Champion Nutrition, Concord with 110 mg of caffeine	1500 mg/L taurine	5 min of light stationary cycling at a self-selected cadence and an additional component of very light stretching and performance of 2–3 light to moderate sets of the squat. Protocol was of 6 sets of the squat exercise with a load equivalent to 75% of subjects predetermined 1-RM.	Area under the curve of resistance-exercise volume was significantly less in baseline than energy drink (10%) and placebo (8.6%). Energy drink (18.4% ± 12.0%) was significantly lower for fatigue rate than baseline (32.9% ± 8.4%)
Ivy et al./the USA [60]	2009	Double-blind, randomized and placebo-controlled	12 (Cyclist athletes)	Both, 27 ± 3 y	Red Bull Energy Drink contains carbonated water, caffeine (80 mg)	Red Bull Energy Drink contains taurine (1000 mg)	Trained cyclists consumed 500 mL of either flavoured placebo or Red Bull^®^ Energy Drink. Performance was measured to 1 hr of cycling at 70% Wmax	Performance improved with energy drink compared with placebo (3.690 ± 64 s vs. 3,.874 ± 93 s, *p* < 0.01)
Ganio et al./the USA [69]	2010	Double-blind, randomized, crossover, repeated measures	15 (Cyclist)	Male, 27 ± 6 y	125 mg/L of caffeine	1920 mg/L of L-taurine	14 male cyclists cycled for 120 min submaximal and then completed a 15-min performance trial. Also, maximal voluntary leg isometric extension	Total work accumulated during performance trial was greater (*p* < 0.05) in energy drink (233 ± 34 KJ) than placebo (205 ± 52KJ) but not in carbohydrate-electrolyte-only solution (225 ± 39 KJ) vs. placebo. MVC (N) declined (*p* < 0.001) from pre to post in placebo (988 ± 213 KJ to 851 ± 191KJ) and carbohydrate-electrolyte-only solution (970 ± 172 KJ to 870 ± 163KJ) but not in energy drink (953 ± 171 KJ to 904 ± 208 KJ). At Minutes 60, 90, 105, and 120 RPE was lower in energy drink than in placebo (*p* < 0.001).
Rahnama et al./Iran [68]	2010	Randomized, placebo controlled, counterbalanced double-blind	10 (Student athletes)	Male, 22 ± 2 y	Red Bull^®^ Energy Drink caffeine (80 mg)	Red Bull^®^ Energy Drink 1000 mg of taurine	Maximal oxygen consumption tests on a treadmill.	Greater value in VO_2_max and time to exhaustion for the Red Bull^®^ and Hype trial compared to placebo (*p* < 0.05)
Sünram-Lea et al./the UK [70]	2012	Double-blind and mixed measures design	81 (Healthy volunteers)	Both, 27 ± 1 y	Two drinks; (1) 50 g glucose and 40 mg caffeine and (2) 10.25 g of fructose/glucose (59% glucose and 41% fructose) and 80 mg caffeine	No Taurine	Range of cognitive tasks, mood, and physiological measures (handgrip test)	An increase in grip strength and improved memory performance after ingestion of the drink containing 50 g glucose and 40 mg caffeine was observed and both active drinks. Improved performance on the information processing task compared to the placebo
Del Coso et al./Spain [62]	2013	Double-blind, placebo controlled and randomized experimental	16 (Rugby-seven athletes)	Female, 23 ± 2 y	(Fure^®^, ProEnergetics, Spain) provide a dose of 3 mg of caffeine per kg of BM.	18.7 mg/kg	Participants performed 15 s maximal jumps test, 6 ×30 m sprint test and 3 rugby matches (running distance)	Fure^®^ increased the ball velocity (19.2 ± 2.1m/s vs 19.7 ± 1.9 m/s, *p* = 0.023), jumping spike (17.9 ± 2.2 m/s vs. 18.8 ± 2.2 m/s, *p* = 0.038) and jump height in the SJ (28.1 ± 3.2 cm vs. 29.4 ± 3.6 cm, *p* = 0.028), CMJ (32.0 ± 4.6 vs. 33.1 ± 4.5 cm, *p* = 0.018), spike jump (43.3 ± 4.7 cm vs. 44.4 ± 5.0 cm, *p* = 0.025), and block jump (35.2 ± 5.1 cm vs. 36.1 ± 5.1 cm, *p* = 0.044).
Lara et al./Spain [13]	2014	Double-blind, placebo controlled, randomized experimental design	18 (Soccer players)	Female, 21 ± 2 y	(Fure^®^, ProEnergetics, Spain)	18.7 mg/kg	Standardized warm-up and CMJ, 7 × 30 m sprint and 2 × 40 m	Fure^®^ increased performance in CMJ (26.6 ± 4.0 cm vs 27.4 ± 3.8 cm; *p* < 0.05), 7 × 30m sprint (24.2 ± 1.6 km/h vs. 24.5 ± 1.7 km/h; *p* < 0.05) and in match simulation 2 × 40m (6.631 ± 1.618 m with the placebo drink and 7.087 ± 1.501 m with the caffeinated energy drink (*p* < 0.05).
Del Coso et al./Spain [18]	2014	Double-blind, placebo controlled, randomized experimental	15 (Volleyball players)	Male, 22 ± 7 y	(Fure^®^, ProEnergetics, Spain)	18.7 mg/kg	Volleyball-specific tests: standing spike test, maximal SJ, maximal CMJ, 15RJ test, and agility T-test	Energy drink increased the spike test (73 ± 9 km/h 75 ± 10 km/h, *p* < 0.05) and jump height in SJ (31.1 ± 4.3 cm vs. 32.7 ± 4.2 cm, *p* < 0.05), CMJ (35.9 ± 4.6 vs. 37.7 ± 4.4 cm, *p* < 0.05), and 15RJ (29.0 ± 4.0 cm vs. 30.5 ± 4.6 cm, *p* < 0.05). The agility test time was significantly reduced with the caffeinated energy drink (10.8 ± 0.7 s vs. 10.3 ± 0.4 s, *p* < 0.05). Players performed successful volleyball actions more frequently (24.6% ± 14.3% vs 34.3% ± 16.5%, *p* < 0.05) compared to placebo.
Abian et al./Spain [66]	2015	Double-blind, placebo controlled and randomized experimental design	15 (Elite badminton players)	Male, 25 ± 7 y	(Fure^®^, ProEnergetics, Spain)	18.7 mg/kg	Handgrip maximal force production, smash jump without and with shuttlecock, SJ, CMJ and the agility *t*-test. 45-min simulated badminton match was played.	Energy drink increased SJ height (34.5 ± 4.7 cm vs. 36.4 ± 4.3 cm; *p* < 0.05), SJ peak power (*p* < 0.05), CMJ (37.7 ± 4.5 vs. 39.5 ± 5.1 cm; *p* < 0.05) and CMJ peak power (*p* < 0.05). An increased number of total impacts was found during the badminton match (7395 ± 1594 impacts vs. 7707 ± 2033 impacts, *p* < 0.05).
Gallo-Salazar et al./Spain [67]	2015	Double-blind, placebo controlled and randomized experimental	14 (Young elite-level tennis players)	Male 16 ± 1 y	(Fure^®^, ProEnergetics, Spain)	18.7 mg/kg	Handgrip-strength test, a maximal-velocity serving test, and an 8 × 15-m sprint test. Were carried out simulated singles match (best of 3 sets).	Energy drink increased handgrip force by 4.2% ± 7.2% (*p* = 0.03) in both hands, the running pace at high intensity (46.7 ± 28.5 vs. 63.3 ± 27.7 km/h, *p* = 0.02), and the number of sprints (12.1 ± 1.7 vs. 13.2 ± 1.7, *p* = 0.05) during the simulated match.
Lara et al./Spain [65]	2015	Double-blind, placebo controlled and randomized experimental	14 (Sprint swimmers)	Male, 20 y	(Fure^®^, ProEnergetics, Spain)	18.7 mg/kg	CMJ, handgrip test, 50 m simulated swimming competition and swim ergometer maximal intensity test.	Energy drink increased the height in the CMJ (49.4 ± 5.3 cm vs. 50.9 ± 5.2 cm, *p* < 0.05), maximal force during the handgrip test with the right hand (481 ± 49 vs. 498 ± 43, *p* < 0.05). Energy drink reduced the time needed to complete the 50 m simulated swimming competition (27.8 ± 3.4 s vs. 27.5 ± 3.2 s, *p* < 0.05) and it increased peak power (273 ± 55 vs. 303 ± 49, *p* < 0.05).
Pérez-López et al./Spain [64]	2015	Double-blind, placebo controlled and randomized experimental	13 (Elite volleyball)	Female, 25 ± 5 y	(Fure^®^, ProEnergetics, Spain)	18.7 mg/kg	Standardized heating and performed standing spike, jumping spike, spike jump, blocking jump, SJ, CMJ, manual dynamometry, and agility *t*-test.	Energy drink increased the ball velocity (19.2 ± 2.1 m/s vs. 19.7 ± 1.9 m/s, *p* = 0.023), jumping spike (17.9 ± 2.2 m/s vs. 18.8 ± 2.2 m/s, *p* = 0.038) and jump height in the SJ (28.1 ± 3.2 cm vs. 29.4 ± 3.6 cm, *p* = 0.028), CMJ (32.0 ± 4.6 cm vs. 33.1 ± 4.5 cm, *p* = 0.018), SJ (43.3 ± 4.7 cm vs. 44.4 ± 5.0 cm, *p* = 0.025), and block jump (35.2 ± 5.1 cm vs. 36.1 ± 5.1 cm, *p* = 0.044).
Quinlivan et al./Australia [58]	2015	Double-blind, crossover	11 (Cyclist and triathletes)	Male, 31.6 ± 6.1 y	Red Bull^®^ Energy Drink contains caffeine (80 mg)	Red Bull^®^ Energy Drink contains taurine (1000 mg)	1 h cycling at 75% peak power output	Red Bull^®^ intake significantly increased sports performance 109 ± 153 s (2.8%, *p* = 0.039) in comparison with placebo 120 ± 172 s (3.1%, *p* = 0.043).
Del Coso et al./Spain [63]	2016	Double-blind, placebo controlled and randomized experimental	13 (Field hockey players)	Male, 23 ± 4 y	(Fure^®^, ProEnergetics, Spain)	18.7 mg/kg	2 × 25 min simulated field hockey game (total distance, distance high intensity, distance low intensity and sprints)	Energy drink reduced the distance covered at moderate-intensity running (793 ± 135 and 712 ± 116, respectively, *p* = 0.03), the distance covered at high-intensity running (303 ± 67 m and 358 ± 117 m, *p* = 0.05) and sprinting (85 ± 41 m and 117 ± 55 m, respectively, *p* = 0.02) in comparison with placebo.
Jacobson et al./the USA [75]	2018	Randomized, double-blind and placebo-controlled	36 (Healthy volunteers)	Both, 23 ± 2 y	Energy drink with 240 mg of caffeine	Energy drink with 200 mg of taurine	3 separate trials of CMJ and isolated forehand stroke with 15 s rest intervals	The energy drink group increased a significant velocity (*p* = 0.05) and W for the forehand stroke, but not for the CMJ, regarding to placebo..
Astley et al./Brazil [72]	2018	Double-blind cross-over randomized	15 (Resistance-trained athletes)	Male, 21 ± 0.3 y	The energy drink with soda water containing caffeine (64 mg/200 mL)	The energy drink containing taurine (800 mg/200 mL)	Maximum repetition test (80% 1-RM) in bench press, unilateral leg extension, handgrip test, standing long jump and repeated sprint ability	Energy Drink intake increased performance compared to the placebo for the number of repetitions in the unilateral knee extension test of the dominant leg (11.5 ± 0.9 reps vs. 9.5 ± 0.8 reps, *p* = 0.001) and bench press (10.2 ± 0.4 reps vs. 8.1 ± 0.5 reps, *p* = 0.01). Increased isometric strength in the hand-grip maximal test in the right (53.7 ± 1.5 kg vs. 47.7 ± 1.6 kg, *p* = 0.02) and left hand (52.9 ± 1.5 kg vs. 45.9 ± 1.3 kg, *p* = 0.02).
Chtourou et al./France [74]	2019	Randomized double blind, placebo-controlled, counterbalanced and crossover	19 (Physical-education students)	Male, 21 ± 1 y	Red Bull^®^ Energy Drink contains carbonated water, caffeine (80 mg)	Red Bull^®^ Energy Drink contains taurine (1000 mg)	During 60 min, the subjects developed visual reaction time, handgrip test and 30-s Wingate	Energy drinks improves peak and mean power output, handgrip force, pre- and post-exercise blood glucose, blood pressure, and vigor, correlated with reduction of fatigue, anxiety and anger. Reductions in reaction times, depression, confusion, fatigue, anger, anxiety, RPE, and affective load scores were observed after energy drink compared to placebo. Energy drinks improves physical performances and reaction times with RPE, affective load, and pre- and post-exercise blood glucose levels.
**No statistically significant positive association**								
Umaña-Alvarado and Moncada-Jiménez/Costa Rica [77]	2005	Double-blind and randomized crossover	11 (Runners or triathletes)	Male, 30 ± 11 y	The commercially available ED provided (for 100 mL) 32 mg/mL of caffeine	The commercially available ED provided (for 100 mL) 400 mg/mL of taurine	Participants completed two 10 km cross country run	No significant differences were found between mean racing times; however, ratings of perceived exertion were significantly lower when participants ingested the energy drink vs placebo (7.02 ± 1.21 vs.8.01 ± 0.75, *p* < 0.05)
Candow et al./Canada [78]	2009	Double-blind, crossover, repeated measures	17 (University students)	Both, 21 ± 4 y	Sugar-free Red Bull^®^ with 2 mg/kg of caffeine	Sugar-free Red Bull^®^ with 25 mg/kg of taurine	Run time-to-exhaustion at 80% VO_2_max treadmill test	No differences in run time-to-exhaustion (Red Bull^®^: 12.6 ± 3.8 min, placebo: 11.8 ± 3.4 min), perceived exertion (Red Bull: 17.1 ± 2.0, placebo: 16.6 ± 1.8), or blood lactate between groups
Dall’Agnol and Souza/Brazil [79]	2009	Double-blind and randomized crossover	22 (Healthy volunteers)	Male, 26 ± 4 y	Energy drink with 160 mg of caffeine	Energy drink with 2000 mg of taurine	Participants completed and incremental test on cycle-ergometer	There was an increase of 10 W with the administration of the experimental drink, without statistical significance (342 ± 40.60 W vs. 332.50 ± 56.83 W, *p* > 0.05)
Hoffman et al./the USA [83]	2009	Randomized double-blind and crossover	12 (Strength-power athletes)	Male, 21 ± 1 y	Redline Extreme^®^ contained 158 mg of caffeine	No taurine	Reaction test and Wingate (20 s Wingate anaerobic power test)	Significant difference in reaction test was seen between energy drink and placebo in both average number of targets struck (55.8 ± 7.4 vs. 51.9 ± 7.4, respectively) and percent of targets struck (71.9 ± 10.5% vs. 66.8 ± 10.9%, respectively). No significant differences between trials were seen in any anaerobic power measure. Subjective feelings of energy (3.5 ± 0.5 vs. 3.1 ± 0.5) and focus (3.8 ± 0.5 vs. 3.3 ± 0.7) were significantly higher during energy drink compared to placebo
Gwacham and Wagner/the USA [12]	2012	Double-blind, randomized and crossover	20 (Football players)	Male, 20 ± 2 y	AdvoCare Spark energy drink contained 120 mg of caffeine	AdvoCare Spark energy drink contained 200 mg of taurine	Sprint performance and anaerobic power	Energy drink did not significantly affect power (3.84, *p* = 0.066) or sprint time (3.06, *p* = 0.097). There was a significant interaction effect between caffeine use and the beverage for sprint times (4.62, *p* = 0.045), as well as for anaerobic power (5.40, *p* = 0.032), indicating a confounding effect.

Abbreviations: 1-RM, one-repetition maximum; 15RJ, 15-s rebound jump test; b/min, beats/minute; BM, body mass; CAF, caffeine; CHO, carbohydrate, CI, confidence interval, CMJ, Countermovement jump; ED, energy drink, ES, effect size; Kg, kilogram; km/h, kilometres/hour; L, liter; L/min; liter/minute; m, meters; m/s, meters/second, mg, milligram; mg/kg, milligram/kilogram, mg/L, milligram/liter, mL, milliliters; min, minutes; mmol/L, millimole/liter; MVC, maximal voluntary contraction, N, Newton, PLA, placebo; RPE, rating of perceived exertions; seconds; SJ, squat jump, VO_2_max, maximal oxygen consumption; W, Watts; y, years.

**Table 2:** Characteristics of studies referring to the association between energy drinks and genetics.

Statistically Significant Positive Association							
First Author/Country/	Year	Study Design	Sample (n)	Gender/Age/Mean Age	Caffeine dosage	Exercise Performance test	Result
Womack et al./the USA [105]	2012	Randomly, double-blind, placebo-controlled trial	35 (Trained cyclist)	Male, 25.0 ± 7.3 y	6 mg/Kg BM of anhydrous caffeine or a placebo	Simulated 40-km time trials on a cycle ergometer	Caffeine supplementation reduced 40-kilometre time (*p* < 0.05) in AA homozygotes (4.9%; caffeine = 72.4 ± 4.2 min, placebo = 76.1 ± 5.8 min) compared to C allele carriers (1.8%; caffeine = 70.9 ± 4.3 min, placebo = 72.2 ± 4.2 min)
Guest et al./Canada [29]	2018	Split-plot randomized, double-blinded, placebo-controlled	101 (Competitive athletes)	Male, 25 ±4 y	0–2–4 mg/Kg caffeine	10-km cycling time trial	AA genotype decreased time 4.8% at 2 mg/Kg (17.0 ± 0.3 vs. 17.8 ± 0.4 min, *p* = 0.0005) and 6.8% at 4 mg/Kg (16.6 ± 0.3 vs. 17.8 ± 0.4 min, *p* < 0.0001). CC genotype, 4 mg/Kg increased cycling time 13.7% (20.8 ± 0.8 vs. 18.3 ± 0.5 min, *p* = 0.04). Among AA/CC with the AC genotype time decreased 4.8% at 2 mg/Kg (17.0 ± 0.3 vs. 17.8 ± 0.4 min, *p* = 0.0005) and 6.8% at 4 mg/Kg (16.6 ± 0.3 vs. 17.8 ± 0.4 min, *p* < 0.0001). Significant (*p* < 0.0001) caffeine-gene interaction was observed. 4 mg/Kg caffeine decreased cycling time by 3% versus placebo (17.6 ± 0.1 vs. 18.1 ± 0.1 min, *p* = 0.01)
Puente et al./Spain [30]	2018	Case-control ecological experimental	19 (Elite basketball players)	Both, 26.7 ± 3.5 y	3 mg/Kg of caffeine	Abalakov jump test followed by the CODAT test. 20-min simulated basketball game	Caffeine intake increased Abalakov jump height by a mean of 2.9 ± 3.6% in AA homozygotes (*p* = 0.03) while this effect did not reach statistical significance for C-allele carriers (2.3 ± 6.8%, *p* = 0.33). The number of impacts during the simulated game also increased in both AA homozygotes (4.1 ± 5.3%, *p* = 0.02) and C-allele carriers (3.3 ± 3.2%, *p* = 0.01).
Carswell et al./the UK [108]	2020	Double-blind, placebo-controlled crossover	18 (Health adults)	Both, 24 ± 4 y	3 mg/Kg of caffeine	15-min cycling time trial and cognitive performance PVT pre, 50 and 95-min post-supplementation)	Caffeine enhanced exercise performance (*p* < 0.001), but effects were not different between participants with ADORA2A ‘high metabolizers’ vs. ‘low’ sensitivity genotype (+6.4 ± 5.8% vs. +8.2 ± 6.8%), or CYP1A2 ‘fast metabolizers’ vs. ‘slow’ metabolism genotype (+7.2 ± 5.9 vs. +7.0 ± 6.7%, *p* > 0.05)
Grgic et al./Australia [28]	2020	Double-blind, randomized, crossover	22 (Resistance-trained participants)	Male, 27.0 ± 5.6 AA group; 29.8 ± 3.6 CT/TT group	3 mg/Kg of caffeine	Movement velocity, power output in the bench press, quality, and quantity of performed repetitions in the bench press exercise, vertical jump height in a CMJ test and power output in a Wingate test	Caffeine ingestion enhanced movement velocity and power output across all loads (ES: 0.20–0.61, *p* < 0.05, the quality and quantity of performed repetitions with 85% of 1RM (ES: 0.27–0.85, *p* < 0.001 for all), vertical jump height (ES: 0.15, *p* = 0.017) and power output in the Wingate test (ES: 0.33–0.44, *p* < 0.05 for all genotypes)
Spineli et al./Brazil [109]	2020	Randomized, crossover and double-blind	100 (Adolescents)	Both, 15 ± 2 y	6 mg/Kg of caffeine	Handgrip strength, vertical jumps, agility test, sit-ups, push-up, and the Yo-Yo intermittent recovery test level 1 (Yo-Yo IR1).	Caffeine enhanced sit-up repetitions (CAF = 37 ± 9; PLA = 35 ± 8) and push-up repetitions (CAF = 26 ± 11; PLA = 24 ± 11) and increased distance covered in Yo-Yo IR1 test (CAF = 1010.4 ± 378.9 m; PLA = 903.2 ± 325.7 m) (*p* < 0.05)
**No statistically significant association**							
Pataky et al./the USA [107]	2016	Randomly counterbalanced, double-blind, placebo-controlled	38 (Recreational trained cyclists)	Male, 21 ±1 y	6 mg/Kg of caffeine Additionally, 25 mL of 1.14% caffeine or placebo solution were mouth rinsed before each time trial	3-km simulated time trials	No association in endurance performance in CYP1A A genotypes vs placebo, but favoring AC genotype (5.1% ± 6.1%, *p* = 0.12) vs placebo
Salinero et al./Spain [106]	2017	Double-blind randomized experimental	21 (Healthy participants)	Both, 29.3 ± 7.7 y	3 mg/Kg of caffeine	30 s Wingate test, visual attention, and side effects	No differences in reaction times between caffeine and placebo conditions (276 ± 31 milliseconds vs. 269 ± 71 milliseconds, *p* = 0.681) between AA homozygotes and C-allele carriers. 31.3% of the C-allele carriers reported increased nervousness after caffeine ingestion, while none of the AA homozygotes reported them. Caffeine ingestion increased peak power (682 ± 140 W vs. 667 ± 137 W, *p* = 0.008) and mean power during the Wingate test (527 ± 111 W vs. 518 ± 111 W, *p* < 0.001)
Davenport et al./the UK [111]	2020	Double-blind, four-treatment, randomly, crossover	13 (Well-trained cyclists)	Both, 28 ± 2 y	200 mg of caffeine, 1600 mg of β-alanine and 1000 mg quercetin	30 min of steady-state exercise on a cycle ergometer followed by a 15-min time trial	Caffeine supplementation appeared optimal for improved performance in a subsequent fatiguing time trial without statistical differences (*p* > 0.05)
Muñoz et al./Spain [19]	2020	Double-blind, placebo-controlled, crossover	31 (Professional handball players)	Both, 23.7 ± 2.8 y	3 mg/Kg of caffeine	CMJ test, a sprint test, an agility test, an isometric handgrip test, and several ball throws	There were no genotype x treatment interactions for CYP1A2 or for ADORA2A (*p* < 0.05), only for the ball throwing from 7 m (*p* = 0.037), higher in CYP1A2 AA homozygotes than in C-allele carriers
Glaister et al./the UK [110]	2020	Randomized, double-blind, placebo controlled	66 (Trained cyclist)	Male, 41.9 ± 8.6 y	5 mg/Kg of BM of caffeine or placebo one hour before performance test	Incremental cycling test, followed by ± 30 min of time-trial	Caffeine reduced the time to complete the time-trial, without effect of genotype (caffeine: 29.7 ± 1.8 min; placebo: 30.8 ± 2.3 min (*p* < 0.05));. During submaximal exercise, caffeine reduced mean heart rate by 2.9 ± 3.7 b/min, and also reduced perceived exertion by 0.5 ± 0.8, and increased blood lactate by 0.29 ± 0.42 mmol/L, respiratory exchange ratio by 0.013 ± 0.032, and minute ventilation by 3.1 ± 6.8 L/min.

Abbreviations: 1-RM, one-repetition maximum; ADORA2A, Adenosine A2a Receptor; b/min, beats/minute; BM, body mass; CAF, caffeine; CMJ, Countermovement jump; CYP1A2, cytochrome P450 1A2; CODAT, Change-of-Direction and Acceleration Test; ES, effect size; Kg, kilogram; L/min; liter/minute; m, meters; mg, milligram; mL, milliliters; min, minutes; mmol/L, millimole/liter; PVT, psychomotor vigilance test; PLA, placebo; VO_2_max, maximal oxygen consumption; W, Watts; y, years.

## Data Availability

No new data were created or analyzed in this study. Data sharing is not applicable to this article.

## References

[1] Ali F., Rehman H., Babayan Z., Stapleton D., Joshi D.D. (2015). Energy drinks and their adverse health effects: A systematic review of the current evidence. Postgrad. Med..

[2] Kumar G., Park S., Onufrak S. (2015). Perceptions about energy drinks are associated with energy drink intake among U.S. youth. Am. J. Health Promot..

[3] Higgins J.P., Babu K., Deuster P.A., Shearer J. (2018). Energy Drinks: A Contemporary Issues Paper. Curr. Sports Med. Rep..

[4] Porrini M., Del Bo’ C. (2016). Ergogenic Aids and Supplements. Front. Horm. Res..

[5] Kerksick C.M., Wilborn C.D., Roberts M.D., Smith-Ryan A., Kleiner S.M., Jäger R., Collins R., Cooke M., Davis J.N., Galvan E. (2018). ISSN exercise & sports nutrition review update: Research & recommendations. J. Int. Soc. Sports Nutr..

[6] Bishop D. (2010). Dietary supplements and team-sport performance. Sports Med..

[7] McDowall J.A. (2007). Supplement use by Young Athletes. J. Sports Sci. Med..

[8] Rawson E.S., Miles M.P., Larson-Meyer D.E. (2018). Dietary Supplements for Health, Adaptation, and Recovery in Athletes. Int. J. Sport Nutr. Exerc. Metab..

[9] Souza D.B., Del Coso J., Casonatto J., Polito M.D. (2017). Acute effects of caffeine-containing energy drinks on physical performance: A systematic review and meta-analysis. Eur. J. Nutr..

[10] Astorino T.A., Roberson D.W. (2010). Efficacy of acute caffeine ingestion for short-term high-intensity exercise performance: A systematic review. J. Strength Cond. Res..

[11] McLellan T.M., Lieberman H.R. (2012). Do energy drinks contain active components other than caffeine?. Nutr. Rev..

[12] Gwacham N., Wagner D.R. (2012). Acute effects of a caffeine-taurine energy drink on repeated sprint performance of American college football players. Int. J. Sport Nutr. Exerc. Metab..

[13] Lara B., Gonzalez-Millán C., Salinero J.J., Abian-Vicen J., Areces F., Barbero-Alvarez J.C., Muñoz V., Portillo L.J., Gonzalez-Rave J.M., Del Coso J. (2014). Caffeine-containing energy drink improves physical performance in female soccer players. Amino Acids.

[14] Del Coso J., Muñoz-Fernández V.E., Muñoz G., Fernández-Elías V.E., Ortega J.F., Hamouti N., Barbero J.C., Muñoz-Guerra J. (2012). Effects of a caffeine-containing energy drink on simulated soccer performance. PLoS ONE.

[15] Astorino T.A., Matera A.J., Basinger J., Evans M., Schurman T., Marquez R. (2012). Effects of red bull energy drink on repeated sprint performance in women athletes. Amino Acids.

[16] Graham T.E. (2001). Caffeine and exercise: Metabolism, endurance and performance. Sports Med..

[17] Prins P.J., Goss F.L., Nagle E.F., Beals K., Robertson R.J., Lovalekar M.T., Welton G.L. (2016). Energy Drinks Improve Five-Kilometer Running Performance in Recreational Endurance Runners. J. Strength Cond. Res..

[18] Del Coso J., Pérez-López A., Abian-Vicen J., Salinero J.J., Lara B., Valadés D. (2014). Enhancing physical performance in male volleyball players with a caffeine-containing energy drink. Int. J. Sports Physiol. Perform..

[19] Muñoz A., López-Samanes Á., Aguilar-Navarro M., Varillas-Delgado D., Rivilla-García J., Moreno-Pérez V., Del Coso J. (2020). Effects of CYP1A2 and ADORA2A Genotypes on the Ergogenic Response to Caffeine in Professional Handball Players. Genes.

[20] Grasser E.K., Miles-Chan J.L., Charrière N., Loonam C.R., Dulloo A.G., Montani J.P. (2016). Energy Drinks and Their Impact on the Cardiovascular System: Potential Mechanisms. Adv. Nutr..

[21] Basrai M., Schweinlin A., Menzel J., Mielke H., Weikert C., Dusemund B., Putze K., Watzl B., Lampen A., Bischoff S.C. (2019). Energy Drinks Induce Acute Cardiovascular and Metabolic Changes Pointing to Potential Risks for Young Adults: A Randomized Controlled Trial. J. Nutr..

[22] Wolk B.J., Ganetsky M., Babu K.M. (2012). Toxicity of energy drinks. Curr. Opin. Pediatr..

[23] Chrysant S.G., Chrysant G.S. (2015). Cardiovascular complications from consumption of high energy drinks: Recent evidence. J. Hum. Hypertens..

[24] Pickering C., Grgic J. (2019). Caffeine and Exercise: What Next?. Sports Med..

[25] Guest N.S., Horne J., Vanderhout S.M., El-Sohemy A. (2019). Sport Nutrigenomics: Personalized Nutrition for Athletic Performance. Front. Nutr..

[26] Southward K., Rutherfurd-Markwick K., Badenhorst C., Ali A. (2018). The Role of Genetics in Moderating the Inter-Individual Differences in the Ergogenicity of Caffeine. Nutrients.

[27] Pickering C., Kiely J. (2018). Are the Current Guidelines on Caffeine Use in Sport Optimal for Everyone? Inter-individual Variation in Caffeine Ergogenicity, and a Move Towards Personalised Sports Nutrition. Sports Med..

[28] Grgic J., Pickering C., Bishop D.J., Schoenfeld B.J., Mikulic P., Pedisic Z. (2020). CYP1A2 genotype and acute effects of caffeine on resistance exercise, jumping, and sprinting performance. J. Int. Soc. Sports Nutr..

[29] Guest N., Corey P., Vescovi J., El-Sohemy A. (2018). Caffeine, CYP1A2 Genotype, and Endurance Performance in Athletes. Med. Sci. Sports Exerc..

[30] Puente C., Abián-Vicén J., Del Coso J., Lara B., Salinero J.J. (2018). The CYP1A2 -163C>A polymorphism does not alter the effects of caffeine on basketball performance. PLoS ONE.

[31] Grgic J., Pickering C., Del Coso J., Schoenfeld B.J., Mikulic P. (2020). CYP1A2 genotype and acute ergogenic effects of caffeine intake on exercise performance: A systematic review. Eur. J. Nutr..

[32] Salinero J.J., Lara B., Abian-Vicen J., Gonzalez-Millán C., Areces F., Gallo-Salazar C., Ruiz-Vicente D., Del Coso J. (2014). The use of energy drinks in sport: Perceived ergogenicity and side effects in male and female athletes. Br. J. Nutr..

[33] Burke L.M. (2008). Caffeine and sports performance. Appl. Physiol. Nutr. Metab..

[34] Mottram D., Chester N., Atkinson G., Goode D. (2008). Athletes’ knowledge and views on OTC medication. Int. J. Sports Med..

[35] Wickham K.A., Spriet L.L. (2018). Administration of Caffeine in Alternate Forms. Sports Med..

[36] Kamimori G.H., Karyekar C.S., Otterstetter R., Cox D.S., Balkin T.J., Belenky G.L., Eddington N.D. (2002). The rate of absorption and relative bioavailability of caffeine administered in chewing gum versus capsules to normal healthy volunteers. Int. J. Pharm..

[37] Campbell B., Wilborn C., La Bounty P., Taylor L., Nelson M.T., Greenwood M., Ziegenfuss T.N., Lopez H.L., Hoffman J.R., Stout J.R. (2013). International Society of Sports Nutrition position stand: Energy drinks. J. Int. Soc. Sports Nutr..

[38] Pasman W.J., van Baak M.A., Jeukendrup A.E., de Haan A. (1995). The effect of different dosages of caffeine on endurance performance time. Int. J. Sports Med..

[39] Polito M.D., Grandolfi K., de Souza D.B. (2019). Caffeine and resistance exercise: The effects of two caffeine doses and the influence of individual perception of caffeine. Eur. J. Sport Sci..

[40] Jenkins N.T., Trilk J.L., Singhal A., O’Connor P.J., Cureton K.J. (2008). Ergogenic effects of low doses of caffeine on cycling performance. Int. J. Sport Nutr. Exerc. Metab..

[41] Southward K., Rutherfurd-Markwick K.J., Ali A. (2018). The Effect of Acute Caffeine Ingestion on Endurance Performance: A Systematic Review and Meta-Analysis. Sports Med..

[42] Bruce C.R., Anderson M.E., Fraser S.F., Stepto N.K., Klein R., Hopkins W.G., Hawley J.A. (2000). Enhancement of 2000-m rowing performance after caffeine ingestion. Med. Sci. Sports Exerc..

[43] Rivers W.H., Webber H.N. (1907). The action of caffeine on the capacity for muscular work. J. Physiol..

[44] Goldstein E.R., Ziegenfuss T., Kalman D., Kreider R., Campbell B., Wilborn C., Taylor L., Willoughby D., Stout J., Graves B.S. (2010). International society of sports nutrition position stand: Caffeine and performance. J. Int. Soc. Sports Nutr..

[45] Killen L.G., Green J.M., O’Neal E.K., McIntosh J.R., Hornsby J., Coates T.E. (2013). Effects of caffeine on session ratings of perceived exertion. Eur. J. Appl. Physiol..

[46] Vicente-Salar N., Santos-Sánchez G., Roche E. (2020). Nutritional Ergogenic Aids in Racquet Sports: A Systematic Review. Nutrients.

[47] Steele D.S., Smith G.L., Miller D.J. (1990). The effects of taurine on Ca2+ uptake by the sarcoplasmic reticulum and Ca2+ sensitivity of chemically skinned rat heart. J. Physiol..

[48] Stapleton P.P., Charles R.P., Redmond H.P., Bouchier-Hayes D.J. (1997). Taurine and human nutrition. Clin. Nutr..

[49] Spriet L.L., Whitfield J. (2015). Taurine and skeletal muscle function. Curr. Opin. Clin. Nutr. Metab. Care.

[50] Hamilton E.J., Berg H.M., Easton C.J., Bakker A.J. (2006). The effect of taurine depletion on the contractile properties and fatigue in fast-twitch skeletal muscle of the mouse. Amino Acids.

[51] Huxtable R.J. (1992). Physiological actions of taurine. Physiol. Rev..

[52] Balshaw T.G., Bampouras T.M., Barry T.J., Sparks S.A. (2013). The effect of acute taurine ingestion on 3-km running performance in trained middle-distance runners. Amino Acids.

[53] Rutherford J.A., Spriet L.L., Stellingwerff T. (2010). The effect of acute taurine ingestion on endurance performance and metabolism in well-trained cyclists. Int. J. Sport Nutr. Exerc. Metab..

[54] Zhang M., Izumi I., Kagamimori S., Sokejima S., Yamagami T., Liu Z., Qi B. (2004). Role of taurine supplementation to prevent exercise-induced oxidative stress in healthy young men. Amino Acids.

[55] Waldron M., Patterson S.D., Tallent J., Jeffries O. (2018). The Effects of an Oral Taurine Dose and Supplementation Period on Endurance Exercise Performance in Humans: A Meta-Analysis. Sports Med..

[56] Huxtable R., Bressler R. (1974). Taurine concentrations in congestive heart failure. Science.

[57] Hoyte C.O., Albert D., Heard K.J. (2013). The use of energy drinks, dietary supplements, and prescription medications by United States college students to enhance athletic performance. J. Community Health.

[58] Quinlivan A., Irwin C., Grant G.D., Anoopkumar-Dukie S., Skinner T., Leveritt M., Desbrow B. (2015). The effects of Red Bull energy drink compared with caffeine on cycling time-trial performance. Int. J. Sports Physiol. Perform..

[59] Cureton K.J., Warren G.L., Millard-Stafford M.L., Wingo J.E., Trilk J., Buyckx M. (2007). Caffeinated sports drink: Ergogenic effects and possible mechanisms. Int. J. Sport Nutr. Exerc. Metab..

[60] Ivy J.L., Kammer L., Ding Z., Wang B., Bernard J.R., Liao Y.H., Hwang J. (2009). Improved cycling time-trial performance after ingestion of a caffeine energy drink. Int. J. Sport Nutr. Exerc. Metab..

[61] Kovacs E.M., Stegen J., Brouns F. (1998). Effect of caffeinated drinks on substrate metabolism, caffeine excretion, and performance. J. Appl. Physiol. (1985).

[62] Del Coso J., Portillo J., Muñoz G., Abián-Vicén J., Gonzalez-Millán C., Muñoz-Guerra J. (2013). Caffeine-containing energy drink improves sprint performance during an international rugby sevens competition. Amino Acids.

[63] Del Coso J., Portillo J., Salinero J.J., Lara B., Abian-Vicen J., Areces F. (2016). Caffeinated Energy Drinks Improve High-Speed Running in Elite Field Hockey Players. Int. J. Sport Nutr. Exerc. Metab..

[64] Pérez-López A., Salinero J.J., Abian-Vicen J., Valadés D., Lara B., Hernandez C., Areces F., González C., Del Coso J. (2015). Caffeinated energy drinks improve volleyball performance in elite female players. Med. Sci. Sports Exerc..

[65] Lara B., Ruiz-Vicente D., Areces F., Abián-Vicén J., Salinero J.J., Gonzalez-Millán C., Gallo-Salazar C., Del Coso J. (2015). Acute consumption of a caffeinated energy drink enhances aspects of performance in sprint swimmers. Br. J. Nutr..

[66] Abian P., Del Coso J., Salinero J.J., Gallo-Salazar C., Areces F., Ruiz-Vicente D., Lara B., Soriano L., Muñoz V., Abian-Vicen J. (2015). The ingestion of a caffeinated energy drink improves jump performance and activity patterns in elite badminton players. J. Sports Sci..

[67] Gallo-Salazar C., Areces F., Abián-Vicén J., Lara B., Salinero J.J., Gonzalez-Millán C., Portillo J., Muñoz V., Juarez D., Del Coso J. (2015). Enhancing physical performance in elite junior tennis players with a caffeinated energy drink. Int. J. Sports Physiol. Perform..

[68] Rahnama N., Gaeini A.A., Kazemi F. (2010). The effectiveness of two energy drinks on selected indices of maximal cardiorespiratory fitness and blood lactate levels in male athletes. J. Res. Med. Sci..

[69] Ganio M.S., Klau J.F., Lee E.C., Yeargin S.W., McDermott B.P., Buyckx M., Maresh C.M., Armstrong L.E. (2010). Effect of various carbohydrate-electrolyte fluids on cycling performance and maximal voluntary contraction. Int. J. Sport Nutr. Exerc. Metab..

[70] Sünram-Lea S.I., Owen-Lynch J., Robinson S.J., Jones E., Hu H. (2012). The effect of energy drinks on cortisol levels, cognition and mood during a fire-fighting exercise. Psychopharmacology.

[71] Ratamess N.A., Hoffman J.R., Ross R., Shanklin M., Faigenbaum A.D., Kang J. (2007). Effects of an amino acid/creatine energy supplement on the acute hormonal response to resistance exercise. Int. J. Sport Nutr. Exerc. Metab..

[72] Astley C., Souza D.B., Polito M.D. (2018). Acute Specific Effects of Caffeine-containing Energy Drink on Different Physical Performances in Resistance-trained Men. Int. J. Exerc. Sci..

[73] Alford C., Cox H., Wescott R. (2001). The effects of red bull energy drink on human performance and mood. Amino Acids.

[74] Chtourou H., Trabelsi K., Ammar A., Shephard R.J., Bragazzi N.L. (2019). Acute Effects of an “Energy Drink” on Short-Term Maximal Performance, Reaction Times, Psychological and Physiological Parameters: Insights from a Randomized Double-Blind, Placebo-Controlled, Counterbalanced Crossover Trial. Nutrients.

[75] Jacobson B.H., Hester G.M., Palmer T.B., Williams K., Pope Z.K., Sellers J.H., Conchola E.C., Woolsey C., Estrada C. (2018). Effect of Energy Drink Consumption on Power and Velocity of Selected Sport Performance Activities. J. Strength Cond. Res..

[76] Thomas C.J., Rothschild J., Earnest C.P., Blaisdell A. (2019). The Effects of Energy Drink Consumption on Cognitive and Physical Performance in Elite League of Legends Players. Sports.

[77] Umaña-Alvarado M., Moncada-Jiménez J. (2005). Consumption of an “energy drink” does not improve aerobic performance in male athletes. Int. J. Appl. Sports Sci..

[78] Candow D.G., Kleisinger A.K., Grenier S., Dorsch K.D. (2009). Effect of sugar-free Red Bull energy drink on high-intensity run time-to-exhaustion in young adults. J. Strength Cond. Res..

[79] Agnol T.D., Souza P.F.A.D. (2009). Efeitos fisiológicos agudos da taurina contida em uma bebida energética em indivíduos fisicamente ativos. Revista Brasileira Med. Esporte.

[80] Kammerer M., Jaramillo J.A., García A., Calderón J.C., Valbuena L.H. (2014). Effects of energy drink major bioactive compounds on the performance of young adults in fitness and cognitive tests: A randomized controlled trial. J. Int. Soc. Sports Nutr..

[81] Pettitt R.W., Niemeyer J.D., Sexton P.J., Lipetzky A., Murray S.R. (2013). Do the noncaffeine ingredients of energy drinks affect metabolic responses to heavy exercise?. J. Strength Cond. Res..

[82] Nelson M.T., Biltz G.R., Dengel D.R. (2014). Cardiovascular and ride time-to-exhaustion effects of an energy drink. J. Int. Soc. Sports Nutr..

[83] Hoffman J.R., Kang J., Ratamess N.A., Hoffman M.W., Tranchina C.P., Faigenbaum A.D. (2009). Examination of a pre-exercise, high energy supplement on exercise performance. J. Int. Soc. Sports Nutr..

[84] Eckerson J.M., Bull A.J., Baechle T.R., Fischer C.A., O’Brien D.C., Moore G.A., Yee J.C., Pulverenti T.S. (2013). Acute ingestion of sugar-free red bull energy drink has no effect on upper body strength and muscular endurance in resistance trained men. J. Strength Cond. Res..

[85] Goel V., Manjunatha S., Pai K.M. (2014). Effect of red bull energy drink on auditory reaction time and maximal voluntary contraction. Indian J. Physiol. Pharmacol..

[86] Cappelletti S., Piacentino D., Sani G., Aromatario M. (2015). Caffeine: Cognitive and physical performance enhancer or psychoactive drug?. Curr. Neuropharmacol..

[87] Reissig C.J., Strain E.C., Griffiths R.R. (2009). Caffeinated energy drinks—A growing problem. Drug Alcohol Depend..

[88] Petit A., Levy F., Lejoyeux M., Reynaud M., Karila L. (2012). Energy drinks: An unknown risk. Rev. Prat..

[89] Holmgren P., Nordén-Pettersson L., Ahlner J. (2004). Caffeine fatalities—four case reports. Forensic Sci. Int..

[90] Kerrigan S., Lindsey T. (2005). Fatal caffeine overdose: Two case reports. Forensic Sci. Int..

[91] Rudolph T., Knudsen K. (2010). A case of fatal caffeine poisoning. Acta Anaesthesiol. Scand..

[92] Sepkowitz K.A. (2013). Energy drinks and caffeine-related adverse effects. JAMA.

[93] Nawrot P., Jordan S., Eastwood J., Rotstein J., Hugenholtz A., Feeley M. (2003). Effects of caffeine on human health. Food Addit. Contam..

[94] Dhar R., Stout C.W., Link M.S., Homoud M.K., Weinstock J., Estes N.A. (2005). Cardiovascular toxicities of performance-enhancing substances in sports. Mayo Clin. Proc..

[95] Shekelle P.G., Hardy M.L., Morton S.C., Maglione M., Mojica W.A., Suttorp M.J., Rhodes S.L., Jungvig L., Gagné J. (2003). Efficacy and safety of ephedra and ephedrine for weight loss and athletic performance: A meta-analysis. JAMA.

[96] Williams A.D., Cribb P.J., Cooke M.B., Hayes A. (2008). The effect of ephedra and caffeine on maximal strength and power in resistance-trained athletes. J. Strength Cond. Res..

[97] Haller C.A., Jacob P., Benowitz N.L. (2004). Enhanced stimulant and metabolic effects of combined ephedrine and caffeine. Clin. Pharmacol. Ther..

[98] Fenster L., Quale C., Hiatt R.A., Wilson M., Windham G.C., Benowitz N.L. (1998). Rate of caffeine metabolism and risk of spontaneous abortion. Am. J. Epidemiol..

[99] Rétey J.V., Adam M., Honegger E., Khatami R., Luhmann U.F., Jung H.H., Berger W., Landolt H.P. (2005). A functional genetic variation of adenosine deaminase affects the duration and intensity of deep sleep in humans. Proc. Natl. Acad. Sci. USA.

[100] Rétey J.V., Adam M., Khatami R., Luhmann U.F., Jung H.H., Berger W., Landolt H.P. (2007). A genetic variation in the adenosine A2A receptor gene (ADORA2A) contributes to individual sensitivity to caffeine effects on sleep. Clin. Pharmacol. Ther..

[101] Rogers P.J., Hohoff C., Heatherley S.V., Mullings E.L., Maxfield P.J., Evershed R.P., Deckert J., Nutt D.J. (2010). Association of the anxiogenic and alerting effects of caffeine with ADORA2A and ADORA1 polymorphisms and habitual level of caffeine consumption. Neuropsychopharmacology.

[102] Erblang M., Drogou C., Gomez-Merino D., Metlaine A., Boland A., Deleuze J.F., Thomas C., Sauvet F., Chennaoui M. (2019). The Impact of Genetic Variations in ADORA2A in the Association between Caffeine Consumption and Sleep. Genes.

[103] Iio W., Matsukawa N., Tsukahara T., Toyoda A. (2012). The effects of oral taurine administration on behavior and hippocampal signal transduction in rats. Amino Acids.

[104] Horvath D.M., Murphy R.M., Mollica J.P., Hayes A., Goodman C.A. (2016). The effect of taurine and β-alanine supplementation on taurine transporter protein and fatigue resistance in skeletal muscle from mdx mice. Amino Acids.

[105] Womack C.J., Saunders M.J., Bechtel M.K., Bolton D.J., Martin M., Luden N.D., Dunham W., Hancock M. (2012). The influence of a CYP1A2 polymorphism on the ergogenic effects of caffeine. J. Int. Soc. Sports Nutr..

[106] Salinero J.J., Lara B., Ruiz-Vicente D., Areces F., Puente-Torres C., Gallo-Salazar C., Pascual T., Del Coso J. (2017). CYP1A2 Genotype Variations Do Not Modify the Benefits and Drawbacks of Caffeine during Exercise: A Pilot Study. Nutrients.

[107] Pataky M.W., Womack C.J., Saunders M.J., Goffe J.L., D’Lugos A.C., El-Sohemy A., Luden N.D. (2016). Caffeine and 3-km cycling performance: Effects of mouth rinsing, genotype, and time of day. Scand. J. Med. Sci. Sports.

[108] Carswell A.T., Howland K., Martinez-Gonzalez B., Baron P., Davison G. (2020). The effect of caffeine on cognitive performance is influenced by CYP1A2 but not ADORA2A genotype, yet neither genotype affects exercise performance in healthy adults. Eur. J. Appl. Physiol..

[109] Spineli H., Pinto M.P., Dos Santos B.P., Lima-Silva A.E., Bertuzzi R., Gitaí D.L.G., de Araujo G.G. (2020). Caffeine improves various aspects of athletic performance in adolescents independent of their 163 C > A CYP1A2 genotypes. Scand. J. Med. Sci. Sports.

[110] Glaister M., Chopra K., Pereira De Sena A., Sternbach C., Morina L., Mavrommatis Y. (2020). Caffeine, exercise physiology, and time-trial performance: No effect of ADORA2A or CYP1A2 genotypes. Appl. Physiol. Nutr. Metab..

[111] Davenport A.D., Jameson T.S.O., Kilroe S.P., Monteyne A.J., Pavis G.F., Wall B.T., Dirks M.L., Alamdari N., Mikus C.R., Stephens F.B. (2020). A Randomised, Placebo-Controlled, Crossover Study Investigating the Optimal Timing of a Caffeine-Containing Supplement for Exercise Performance. Sports Med. Open.

[112] Higgins J.P., Tuttle T.D., Higgins C.L. (2010). Energy beverages: Content and safety. Mayo Clin. Proc..

[113] Benowitz N.L. (1990). Clinical pharmacology of caffeine. Annu Rev. Med..

[114] Davis J.M., Zhao Z., Stock H.S., Mehl K.A., Buggy J., Hand G.A. (2003). Central nervous system effects of caffeine and adenosine on fatigue. Am. J. Physiol. Regul. Integr. Comp. Physiol..

[115] Bazzucchi I., Felici F., Montini M., Figura F., Sacchetti M. (2011). Caffeine improves neuromuscular function during maximal dynamic exercise. Muscle Nerve.

[116] McPherson P.S., Kim Y.K., Valdivia H., Knudson C.M., Takekura H., Franzini-Armstrong C., Coronado R., Campbell K.P. (1991). The brain ryanodine receptor: A caffeine-sensitive calcium release channel. Neuron.

[117] Van Soeren M.H., Graham T.E. (1998). Effect of caffeine on metabolism, exercise endurance, and catecholamine responses after withdrawal. J. Appl. Physiol. (1985).

[118] Gutiérrez-Hellín J., Del Coso J. (2018). Effects of p-Synephrine and Caffeine Ingestion on Substrate Oxidation during Exercise. Med. Sci. Sports Exerc..

[119] Portillo J., Del Coso J., Abián-Vicén J. (2017). Effects of Caffeine Ingestion on Skill Performance During an International Female Rugby Sevens Competition. J. Strength Cond. Res..

[120] Watson E.J., Coates A.M., Kohler M., Banks S. (2016). Caffeine Consumption and Sleep Quality in Australian Adults. Nutrients.

[121] Curran C.P., Marczinski C.A. (2017). Taurine, caffeine, and energy drinks: Reviewing the risks to the adolescent brain. Birth Defects Res..

[122] Pickering C., Kiely J. (2019). What Should We Do About Habitual Caffeine Use in Athletes?. Sports Med..

[123] Pallarés J.G., Fernández-Elías V.E., Ortega J.F., Muñoz G., Muñoz-Guerra J., Mora-Rodríguez R. (2013). Neuromuscular responses to incremental caffeine doses: Performance and side effects. Med. Sci. Sports Exerc..

[124] Nowak D., Gośliński M., Nowatkowska K. (2018). The Effect of Acute Consumption of Energy Drinks on Blood Pressure, Heart Rate and Blood Glucose in the Group of Young Adults. Int. J. Environ. Res. Public Health.

[125] Hajsadeghi S., Mohammadpour F., Manteghi M.J., Kordshakeri K., Tokazebani M., Rahmani E., Hassanzadeh M. (2016). Effects of energy drinks on blood pressure, heart rate, and electrocardiographic parameters: An experimental study on healthy young adults. Anatol. J. Cardiol..

[126] Miles-Chan J.L., Charrière N., Grasser E.K., Montani J.P., Dulloo A.G. (2015). The blood pressure-elevating effect of Red Bull energy drink is mimicked by caffeine but through different hemodynamic pathways. Physiol. Rep..

[127] Grgic J., Pickering C., Bishop D.J., Del Coso J., Schoenfeld B.J., Tinsley G.M., Pedisic Z. (2020). ADOR2A C Allele Carriers Exhibit Ergogenic Responses to Caffeine Supplementation. Nutrients.

[128] Martins G.L., Guilherme J., Ferreira L.H.B., de Souza-Junior T.P., Lancha A.H. (2020). Caffeine and Exercise Performance: Possible Directions for Definitive Findings. Front. Sports Act. Living.

[129] Pickering C. (2019). Caffeine, CYP1A2 genotype, and sports performance: Is timing important?. Ir. J. Med. Sci..

[130] Bell D.G., McLellan T.M. (2002). Exercise endurance 1, 3, and 6 h after caffeine ingestion in caffeine users and nonusers. J. Appl. Physiol. (1985).

[131] Ahmetov I.I., Egorova E.S., Gabdrakhmanova L.J., Fedotovskaya O.N. (2016). Genes and Athletic Performance: An Update. Med. Sport Sci..

[132] Joyner M.J. (2019). Genetic Approaches for Sports Performance: How Far Away Are We?. Sports Med..

[133] Varillas Delgado D., Telleria Orriols J.J., Martin Saborido C. (2019). Liver-Metabolizing Genes and Their Relationship to the Performance of Elite Spanish Male Endurance Athletes; a Prospective Transversal Study. Sports Med. Open.

